# Modeling of neotame and fructose thermochemistry: Comparison with mono and divalent metal ions by Computational and experimental approach

**DOI:** 10.1038/s41598-019-54626-9

**Published:** 2019-12-05

**Authors:** Deepali Sharma, Suvardhan Kanchi, Ayyappa Bathinapatla, Inamuddin, Abdullah M. Asiri

**Affiliations:** 1 Department of Pharmaceutical Sciences, University of KwaZulu-Natal, Durban, 4000 South Africa; 2 Department of Chemistry, Faculty of Applied Science, Durban University of Technology, Durban, 4000 South Africa; 3 Chemistry Department, Faculty of Science, King Abdulaziz University, Jeddah, 21589 Saudi Arabia; 4 Centre of Excellence for Advanced Materials Research, King Abdulaziz University, Jeddah, 21589 Saudi Arabia; 5 Advanced Functional Materials Laboratory, Department of Applied Chemistry, Faculty of Engineering and Technology, Aligarh Muslim University, Aligarh, 202 002 India

**Keywords:** Bioanalytical chemistry, Composites

## Abstract

The metal complexes can demonstrate various interesting biological activities in the human body. However, the role of certain metal ions for specific cell activities is still subject to debate. This study is aimed at comparing the thermochemical properties of neotame (artificial sweetener) and α, β-fructose in gas phase and water medium. The interaction of α and β-fructose, neotame with monovalent and divalent metal ions was studied and comprehended by density functional theory (DFT) using B3LYP functional, 6–311 + G (d, p) and D3 basis set. Metal ion affinities (MIA) values depicted that ionic radius of metal ions played an important role in the interaction of α, β-fructose and neotame. The ∆G parameter was calculated to predict and understand the interaction of metal ions with α and β-fructose, neotame. The results suggested that the presence of hydroxyl groups and oxygen atoms in sugar molecules acted as preferred sites for the binding and interaction of mono and divalent ions. For the first time computational study has been introduced in the present study to review the progress in the application of metal binding with sugar molecules especially with neotame. Moreover, voltammetric behaviour of neotame-Zn^2+^ was studied using cyclic and differential pulse voltammetry. The obtained results suggest that the peak at −1.13 V is due to the reduction of Zn^2+^ in 0.1 M phosphate buffer medium at pH 5.5. Whereas, addition of 6-fold higher concentration of neotame to the ZnCl_2_.2H_2_O resulted in a new irreversible cathodic peak at −0.83, due to the reduction of neotame-Zn^2+^ complex. The Fourier transform infrared spectroscopy (FTIR) results indicates that the β-amino group (-NH) and carboxyl carbonyl (-C = O) groups of neotame is participating in the chelation process, which is further supported by DFT studies. The findings of this study identify the efficient chelation factors as major contributors into metal ion affinities, with promising possibilities to determine important biological processes in cell wall and glucose transmembrane transport.

## Introduction

Neotame, (3 S)-3-(3,3-Dimethylbutylamino)-4-[[(2 S)-1-methoxy-1-oxo-3-phenylpropan-2-yl amino]-4-oxobutanoic acid is an artificial sweetener and chemically synthesized from aspartame. It has similar physical properties as aspartame in terms of sweetness and comparable with sucrose without a metallic or bitter after taste at high concentrations. Neotame has benefits over aspartame with pH stability at neutral medium which enables its use in baking, no risk associated with the phenylketonuria individuals and being a cost effective^1–3^. It is significant to record that the neotame may be 13000 times sweeter that sucrose and exhibits temporal flavor profile in water, which is similar to that of aspartame, however the taste release response time is slower than aspartame^3^.

Neotame in the powder form has been highly stable at mild temperature for years and it is pH and temperature dependent^1^. There are no reports available in the literature, stating that neotame is toxic to humans and other mammals, however excess usage of aspartame may cause different health issues such as memory loss, migraine and headache^4^. The studies on complexes of metal ions are very important for biological systems which leads to the clarification of clinical results. The structure of neotame is similar to that of aspartame in terms of functional groups, therefore it acts as a ligand due to the presence of carboxyl and amino groups for metal ion chelation of biological importance^5^. In the recent years, due to the novelty of neotame in the food market, studies on metal ions complexation in solution phase is of growing interest^6–16^. The interaction of sucrose or fructose with different metal ions is vital in biological processes such as metal ions binding to cell wall^17^ and transmembrane transportation of glucose-Na^+^18, which has been established in the gas phase (i.e., intrinsic), thereby resulting in the key information on the chemical properties of the glucose or fructose and various metal ions. To the best of our knowledge, no reports have been available in the literature on the interaction of α or β-fructose and neotame with mono, divalent metal ions in gas phase and water medium (intrinsic) using B3LYP/6–311 + G (d, p)/D3 level of theory.

The selectivity of cell wall depends on the nature, size and charge of the ligand. The cell wall allows the mobility of the ligands and metal ions to cellular fluids from exterior environment. Moreover, the energy barrier for anions and gases to enter through hydrophobic environment is slightly lower compared to the cation ions due to the smaller charge-to-radius ratio and a lesser charge density. Thus, anions pierce directly through the cell wall faster than the cations^18^. To overcome this challenge, present study on the neotame-metal ion complexes were performed to better understand the active functional groups participating in the binding, electrochemical behavior as well as metal ion affinities of complex. The thermochemistry of neotame and α-, β-fructose and neotame of monovalent (Li^+^, Na^+^, K^+^) and divalent (Mg^2+^, Ca^2+^, Fe^2+^, Zn^2+^) metal ions were examined in gas phase and water as solvent and compared with density functional theory (DFT) calculations.

## Experimental

### Computational methods

For α-, β-fructose and neotame, conformers were generated using ConfGen module in Schrodinger software package^19^. The most stable conformer for α, β-fructose and neotame was optimized using the Gaussian 2016 package^20^. The Becke’s 3-parameter exchange functional with Lee-Yang-Parr correlation energy abbreviated as B3LYP together with 6–311 + G (d, p) and D3 basis sets^21,22^ were used for the geometry optimization of the conformers and the metal ion complexes in gas phase and water.

Metal ion affinity (MIA) for α-, β-fructose and neotame was calculated by considering the negative of the enthalpy variation (Δ*H*) for the following ion association process^23^:

$${\rm{X}}+{{\rm{M}}}^{+}\to {[{\rm{X}}-{\rm{M}}]}^{+}$$

where X denotes α-, β-fructose, neotame and M^+^ the particular charged ion. Enthalpy contributions (Δ*H*) were obtained by a thermodynamic analysis at 298 K using the vibrational frequencies at each level of theory.

### Experimental methods

#### Instrumentation

All electrochemical studies were conducted using a 797 VA computrace (Metrohm, Switzerland) equipped with a PC and Computrace 1.3.1 software. The cyclic and differential pulse voltammograms were recorded at room temperature using three electrode system of a glassy carbon, Ag/AgCl and platinum wire as working, reference and auxiliary electrodes, respectively. The neotame-Zn^2+^ complex studies were performed using Varian 800 FTIR Scimitar Series (South Africa) with KBr disks. The absorption measurements of neotame-Zn^2+^ complex was performed using UV 2450 Spectrophotometer (Shimadzu, Japan) within the ultra-violet visible region ranging from 200 to 800 nm.

#### Reagents and solutions

All the reagents used in the present study were of analytical grade and used without further purification. Neotame and zinc chloride dihydrate were obtained from Sigma-Aldrich, Durban, South Africa. Potassium dihydrogen phosphate, dipotassium hydrogen phosphate and hydrochloric acid were obtained from Capital Research Distribution (PTY) LTD, Durban, South Africa. The 99.99% purity nitrogen gas (99.9% purity) was purchased from AFROX, Durban, South Africa. Stock and working standard solutions were prepared freshly every day in ultra-pure deionized water from PURITE (18 MΩ) system.

#### Synthesis of neotame-Zn^2+^ complex

The neotame-Zn^2+^ complex was synthesized as a mononuclear complex as per to the previous studies performed in our laboratory^24^. In a 100 mL beaker, 1 mM of ZnCl_2_.2H_2_O solution was prepared in 20 mL of methanol and added to 2 mM of neotame in 30 mL and pH of the mixture was adjusted to 5.5. Then the solution was magnetically stirred for 4 h at 25 °C and dried at room temperature. The white solid precipitate obtained was subjected to repeated washings with acetone and refrigerated at 4 °C for further purpose.

#### Procedure for electrochemical studies of neotame-Zn^2+^ complex

An aliquot of a standard solution of 1 mM of zinc chloride (5 mL) was added to the voltammetric cell and the solution was purged with 99.99% pure nitrogen gas followed by the addition of 6 mM of neotame (5 mL) and 0.1 M phosphate buffer medium (10 mL, pH 5.5). The voltammograms were recorded at room temperature (25 °C). The potential scans were measured using cyclic and differential pulse voltammetry.

#### Spectroscopic measurements

All spectroscopic measurements were performed with appropriate mole ratio of zinc chloride and neotame aqueous solutions. The changes in color due to the formation of neotame-Zn^2+^ complex was recorded at maximum absorption wavelength within the UV-visible region.

## Results and Discussion

### Computational discussion

**Figure 1:**
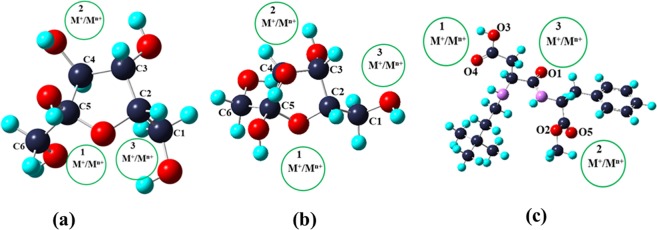
Optimized structures of (**a**) α-fructose (**b**) β-fructose (**c**) neotame.

The first step was to identify the low energy conformations of α-, β-fructose and neotame. For α- and β-fructose, 24 and 29 conformers were found, respectively whereas for neotame 18 conformers were found. The most stable conformer was then optimized using B3LYP/6–311 + G (d, p) and D3 basis set. The optimized structures have been shown in Fig. 1(a–c).

The second step included the coordination of metal ions (Li^+^, Na^+^, K^+^, Mg^2+^, Ca^2+^, Fe^2+^, Zn^2+^) with the optimized structures of α-fructose, β-fructose and neotame using B3LYP/6–311 + G (d, p) and B3LYP/D3 basis set in gas phase as well as in solvent (water) medium. Three positions were considered in each of the optimized molecule where metal ions were coordinated (Fig. 1).

#### Metal ion complexes of α-fructose in gas phase

**Figure 2:**
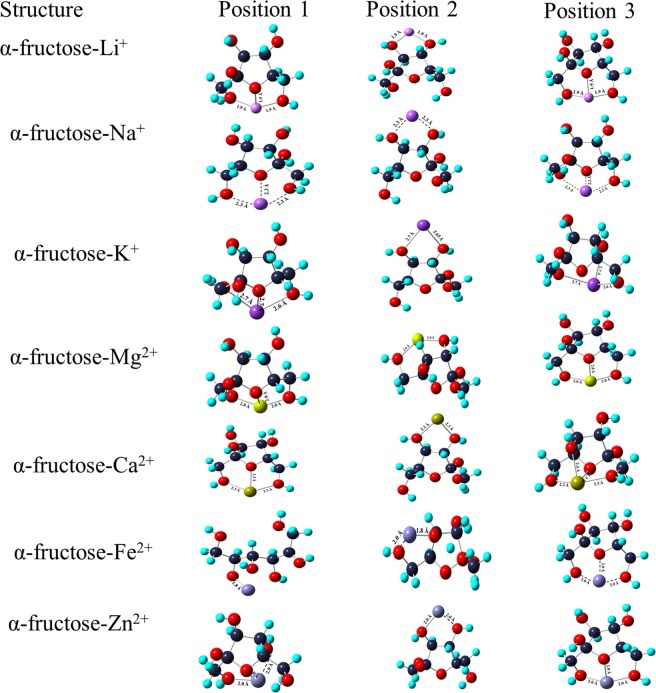
Complexation of α-fructose with mono and divalent ions at three positions using B3LYP/6-311+G(d,p) basis set.

**Figure 3:**
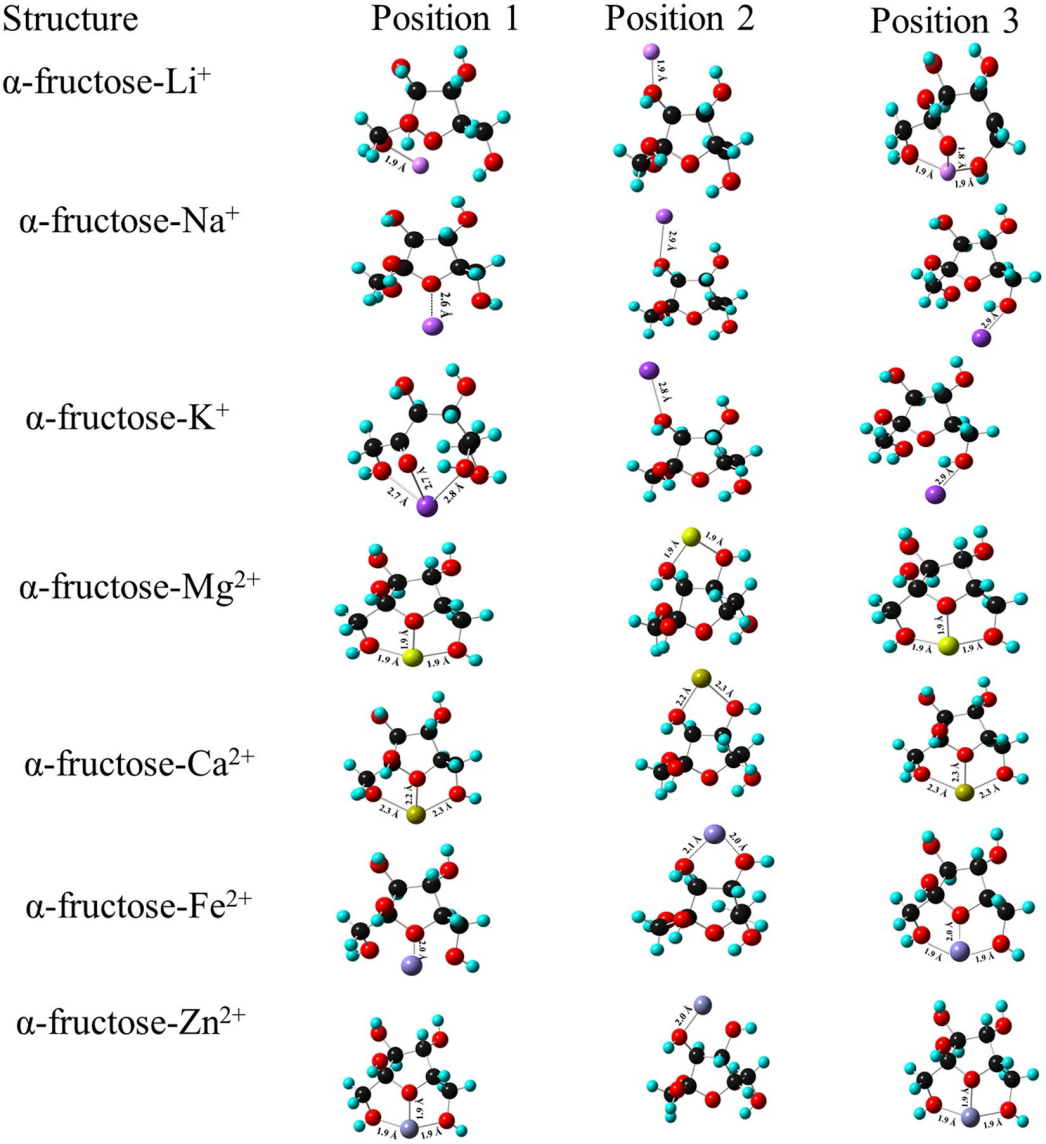
Complexation of α-fructose with mono and divalent ions at three positions using B3LYP/D3 basis set.

The study of interaction of metal ions with fructose are comparable with the metal-saccharide interactions in biological fluids that play a key role in many biological functions^25^. Therefore, the interaction of monovalent and divalent ions with fructose molecule is investigated. Figure 1 represents the ion metalation process of α-fructose in gas phase using B3LYP/6-311 + G (d, p) basis set. Figure 2 depicts the metalation using B3LYP/D3 basis set. Li^+^, Na^+^, and K^+^ ions appears to be tri-coordinated to α-fructose at position 1 and 3 whereas bi-coordinated at position 2 (Fig. 2). Li^+^ is tri-coordinated at position 3 whereas K^+^ is tri-coordinated at position 1 (Fig. 3). From the calculated MIA values, it is observed that metalation of Li^+^ ion is strong at position 3 with an enthalpy energy (Δ*H*) of −79.69 kcal mol^−1^ and −179.47 kcal mol^−1^, respectively. It has been reported in literature that minor changes in the stereochemistry of a saccharide can alter the coordination and metal ion affinity^26^.

The MIA has been found to be high at position 3 for Na^+^ and K^+^ ions with Δ*H* values as −52.08 kcal mol^−1^ and −38.59 kcal mol^−1^, respectively. Among monovalent cations, Li^+^ is more strongly bound to α-fructose. It is observed that the M^+^-O length is ~1.9 Å which is same for all complexes. It has been observed that Na^+^ and K^+^ ions have more affinity for α-fructose at position 1 with Δ*H* values as −136.17 kcal mol^−1^ and −135.54 kcal mol^−1^, respectively. This is also evident from the Na^+^-O and K^+^-O bond lengths of 2.6 Å and 2.7 Å at position 1 as compared to positions 2 and 3 (Fig. 3). The electrostatic and polarization interactions play an important role in determining the geometry of metal saccharide^27^.

The interaction of divalent ions has been shown in Fig. 1. Mg^2+^ ion is tri-coordinated at positions 1 and 3 whereas bicoordinated at position 2. At these positions, there is a change in the stereochemistry of α-fructose on interaction with Mg^2+^ ion (Fig. 2). The H atom of the -OH attached to the C6 of the α-fructose shifted to the equatorial position (positions 1 and 3). The MIA values at these positions is found to be same i.e. −207.71 kcal mol^−1^. The positions 1 and 3 are stable as compared to position 2 where MIA is found to be −163.15 kcal mol^−1^. The Mg^2+^-O bond length at positions 1 and 3 is almost same, 1.96 Å whereas at position 2, the bond length between Mg^2+^ ion and O atoms is ~2.05 Å. Thus, the shortening of the bond length between the metal cation and O atom leads to the formation of the stable complexes. Ca^2+^ ion is tri-coordinated and tetra-coordinated at positions 1 and 3, respectively whereas bicoordinated at position 2. At position 3, the MIA was found to be higher (−166.29 kcal mol^−1^) in comparison to two other positions. Though the bond length between Ca^2+^ and O atom is almost same (2.3 Å) at positions 1, 2 and 3 but the interaction of ion at position 3 leads to the formation of a stable complex. Mg and Ca ions belong to the same group of the periodic table but MIA of Mg^2+^ is higher than that of Ca^2+^. MIA depends on the ionic radius of the ions and since ionic radius of Mg^2+^ is smaller than the Ca^2+^, it coordinates strongly with α-fructose thereby indicating that the size effect plays an important role in the coordination process^28^.

The changes have been observed at positions 1 and 2 on interaction of Fe^2+^ ion with α-fructose. The fructose ring opens at position 1 thus, bringing a change in the structure of a complex. It is also noted that the MIA values is same at position 1 and 2 (−203.31 kcal mol^−1^ and −202.69 kcal mol^−1^). The bond length between Fe^2+^ and O atom is ~1.80 Å at these positions. Fe^2+^ is tri-coordinated at position 3 with MIA value of −218.37 kcal mol^−1^ and Fe^2+^-O bond length of 2.0 Å. In the case of Zn^2+^ ion, a change in the stereochemistry is observed at position 1 with MIA value of −212.09 kcal mol^−1^. At position 2, Zn^2+^ ion is bi-coordinated whereas it is tri-coordinated at position 3 with MIA values of −175.08 kcal mol^−1^ and −225.90 kcal mol^−1^, respectively. The tri-coordination mode leads to stable geometry at position 3. The bond distance between Zn^2+^-O is 2.0 Å in all the positions.

The affinity of divalent cations is observed to be same at position 1 and 3 with MIA values of −210 kcal mol^−1^, −150 kcal mol^−1^, −226.53 kcal mol^−1^ and −229.67 kcal mol^−1^ for Mg^2+^, Ca^2+^, Fe^2+^ and Zn^2+^ ions, respectively.

The MIA has been found to be more stable i.e., monovalent and divalent ions have formed stable complexes using B3LYP/D3 basis set.

#### Metal ion complexes of β-fructose in gas phase

**Figure 4:**
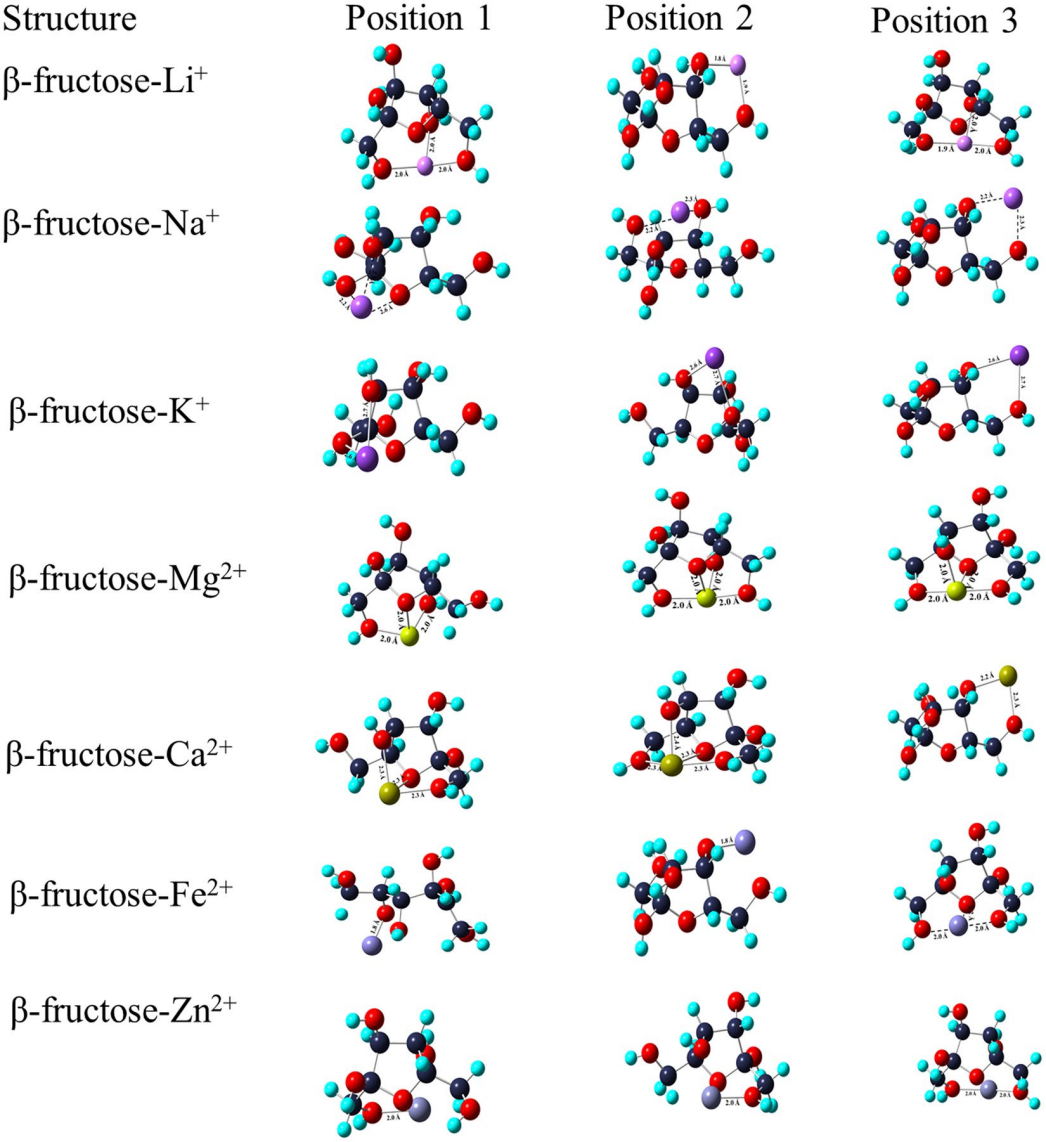
Complexation of β-fructose with mono and divalent ions at three positions using B3LYP/6-311 + G (d, p) basis set.

**Figure 5:**
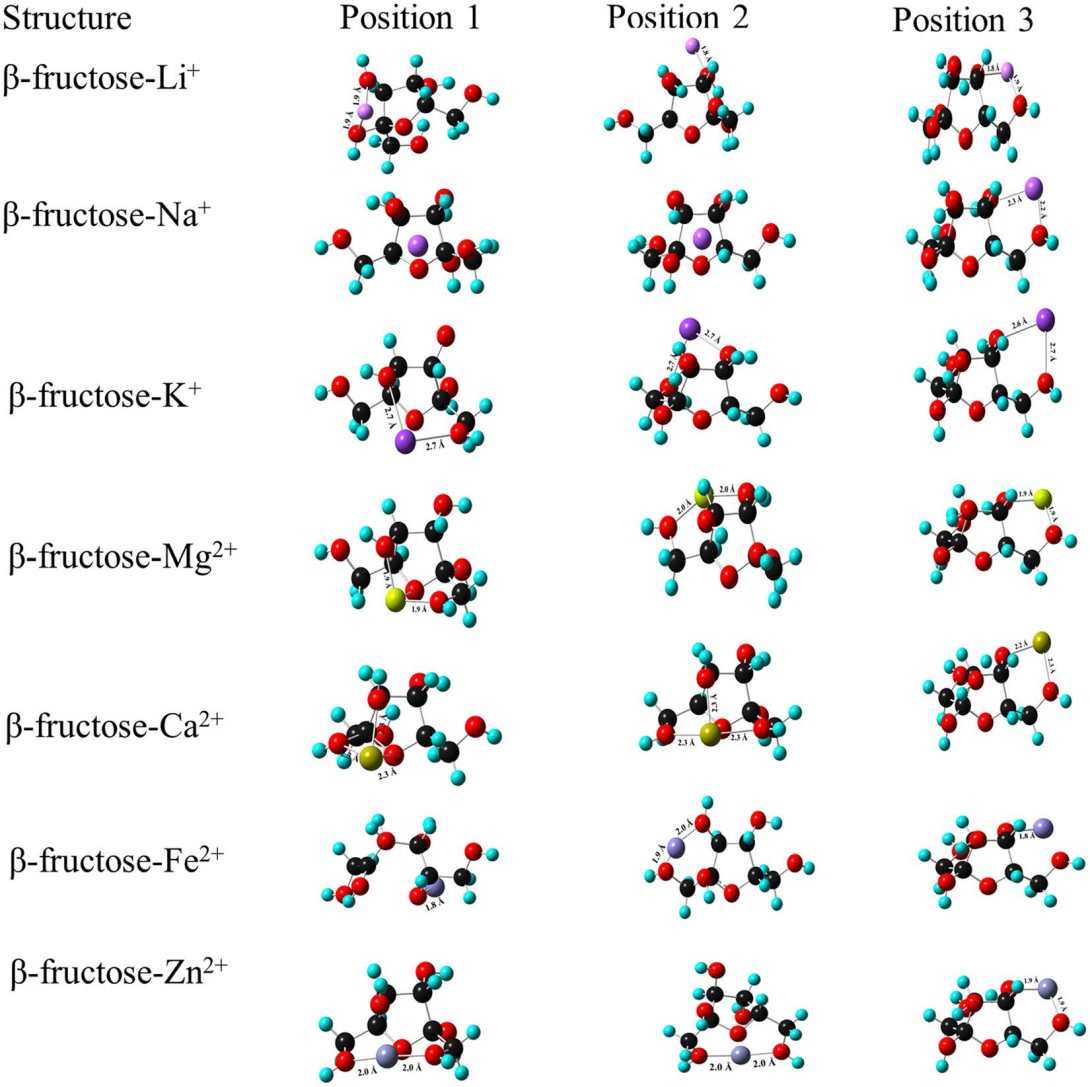
Complexation of β-fructose with mono and divalent ions at three positions using B3LYP/D3 basis set.

The interaction of monovalent and divalent ions with β-fructose has been shown in Figs. 4 and 5. Li^+^ ion is tri-coordinated at positions 1 and 3 whereas bicoordinated at position 2. The MIA values at positions 1 and 3 is found to be −80.32 kcal mol^−1^ which is 12 kcal mol^−1^ higher than that at position 2. The bond length between Li^+^ ion and O atoms is 1.9 Å. In the case of Na^+^ ion, MIA at position 2 and 3 is very close with values of −47.69 kcal mol^−1^ and −48.95 kcal mol^−1^, respectively. The coordination of Na^+^ ion at these positions leads to a stable geometry as compared to that at position 1. The bond length between Na^+^ and O atom is ~2.2 Å. K^+^ ion is bicoordinated at all the positions with MIA higher at position 3 (−16.94 kcal mol^−1^). The MIA value is strongly dependent on the charge-to-size ratio of the ion and its coordination mode with the ligand (fructose). Thus, it is observed that among monovalent ions, Li^+^ ion is strongly coordinated to β-fructose whereas K^+^ ion has low affinity of binding with β-fructose. Moreover, K^+^-O bond length is 2.6 Å which is longer than that of L^+^-O and Na^+^-O bond length.

Mg^2+^ ion is tri-coordinated at position 1 whereas tetra-coordinated at positions 2 and 3. The formation of bond between Mg^2+^ ion and -O, β-fructose atom of the OH attached to C (1) atom has led to the change in the stereochemistry of β-fructose at positions 2 and 3 with MIA value of −231.55 kcal mol^−1^ higher than that at position 1 (−200.18 kcal mol^−1^). Mg^2+^-O bond length is found to be similar at all the positions (~2.0 Å). The interaction of Ca^2+^ ion at position 2 shows major change in the geometry of β-fructose and thus leads to high MIA value of −166.92 kcal mol^−1^. Ca^2+^ ion is tetra-coordinated at this position whereas it is tri-coordinated and bi-coordinated at position 1 and 3, respectively. It is observed that the participation of sugar hydroxyl (-OH) groups in metal-ligand (fructose) interactions affect the MIA values. Moreover, the participation of sugar -OH groups, metal ion-ligand interaction influences electronic distribution of the ring thereby causing a change in the structure of the ring^29^. The bond length between Ca^2+^-O is ~2.3 Å in all the positions. A change in the structure of β-fructose ring is observed on interaction with Fe^2+^ ion. At position 1, there is a ring opening thereby destabilizing the complex and low MIA (−195.16 kcal mol^−1^) is observed. Fe^2+^ ion is bound to O atom of -OH group attached to C4 of the ring at position 2 whereas it is bound to O atom of -OH groups attached to C1, C6 and C4 atoms and O atom of the β-fructose ring at position 3. Thus, position 3 has high MIA value of −246.61 kcal mol^−1^ and is considered to be favourable position for the interaction of Fe^2+^ ion with β-fructose with Fe^2+^-O bond length of 2.0 Å. Zn^2+^ ion interacts strongly at position 3 where it is bicoordinated. O atom of -OH group attached to C1 and C6 of the ring binds to Zn^2+^ ion, thus forming a stable structure with MIA value of −247.87 kcal mol^−1^ higher in comparison to that at positions 1 and 2. At positions 1 and 2, Zn^2+^ ion is coordinated to O atom of hydroxyl group attached to C6 atom and C1 atom, respectively. Zn^2+^-O bond length is 2.0 Å at positions 1, 2 and 3.

The high MIA values have been observed for metal complexes using B3LYP/D3 basis set as compared to B3LYP/6-311 + G (d, p) basis set. Therefore, it can be deduced that B3LYP/D3 basis set leads to the formation of stable metal ion complexes with β-fructose.

#### Metal ion complexes of neotame in gas phase

**Figure 6:**
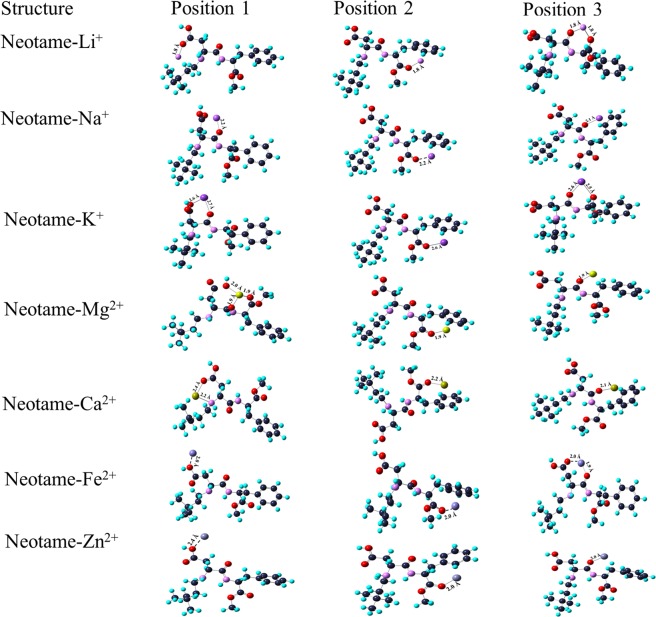
Complexation of neotame with mono and divalent ions at three positions using B3LYP/6-311 + G (d, p) basis set.

**Figure 7:**
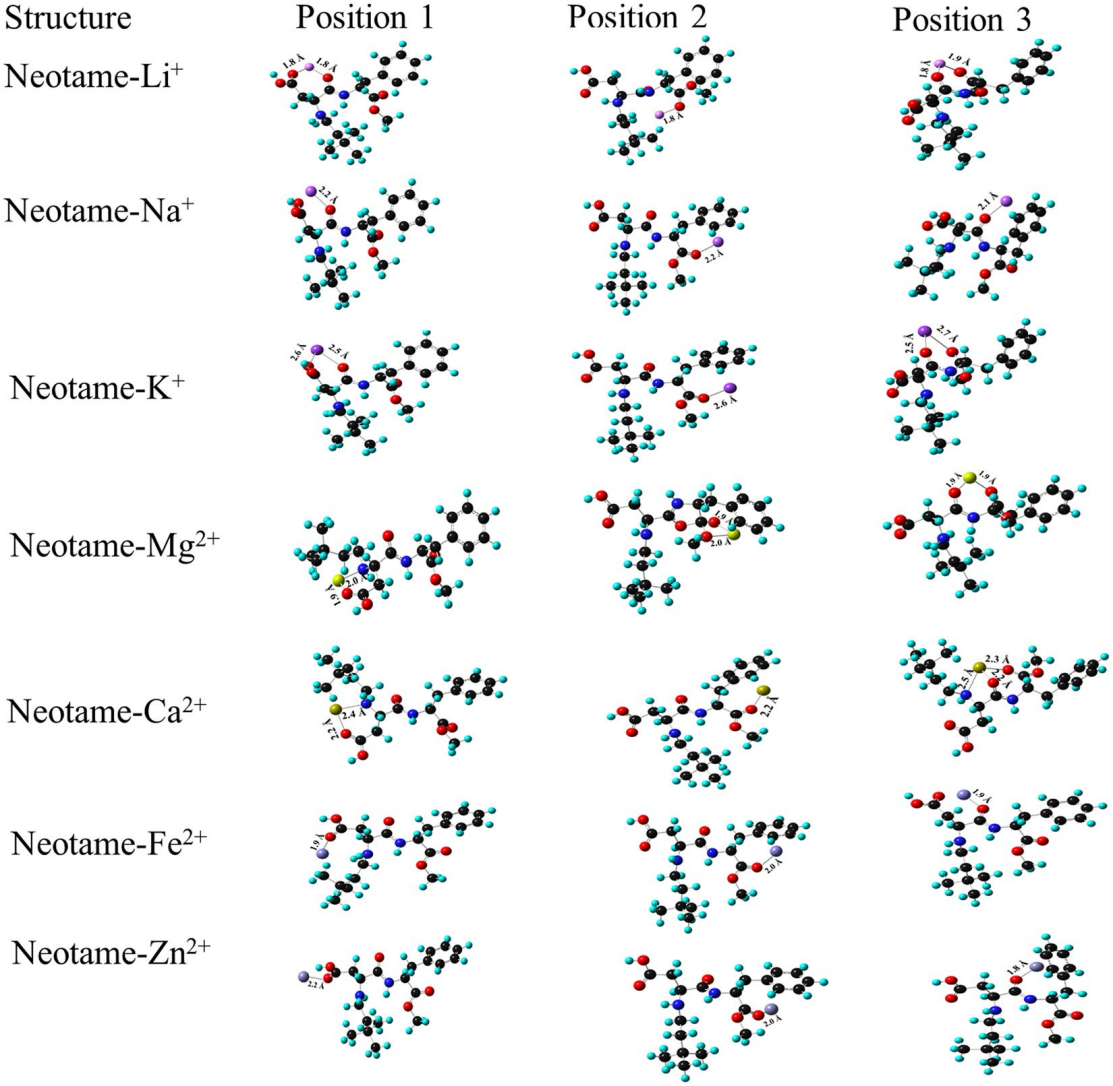
Complexation of neotame with mono and divalent ions at three positions using B3LYP/D3 basis set.

The complexation of monovalent and divalent ions with neotame using 6-311 + G (d, p) and D3 basis set at three different positions have been shown in Figs. 6 and 7. In the case of monovalent ions, Li^+^ ion is bi-coordinated with O (1) and O (5) atoms at position 3. A ring is formed which leads to the formation of a stable complex with MIA of −77.81 kcal mol^−1^. At position 1 and 2, as Li^+^ ion interacts with O atoms, there is distribution of charge around O atoms with MIA values of −49.57 kcal mol^−1^ and −66.52 kcal mol^−1^, respectively. Position 2 has high MIA than position 1 as Li^+^ ion interacts with the phenyl ring of neotame. The interaction of Na^+^ ion with neotame at position 1 and 3 has close MIA values (−53.34 kcal mol^−1^ and −52.08 kcal mol^−1^) whereas MIA is found be high at position 2 (−147.69 kcal mol^−1^). Here, Na^+^ ion interacts strongly with O (5) and phenyl ring of neotame thereby bringing a change in the configuration of the complex. The distance between Na^+^ ion and O atom is 2.2 Å. K^+^ ion is bicoordinated at position 1 and 3 whereas it is coordinated to O atom and phenyl ring at position 2. The MIA is high at position 3 with Δ*H* of −42.04 kcal mol^−1^. Since, the bond length between K^+^ ion and O atom is 2.6 Å, the MIA of neotame with K^+^ ion is least as compared to Li^+^ and Na^+^ ions.

Mg^2+^ ion is bi-coordinated to O (1) and O (5) atoms at position 1 and there is rearrangement of OH group which shifts from C (19) and binds to Mg^2+^ ion thereby leading to high MIA (−229.04 kcal mol^−1^). At position 2, Mg^2+^ ion is coordinated to O (5) and phenyl ring of neotame whereas at position 3, metal ion is coordinated to O (1). As seen in Fig. 6, electrostatic interaction exists between metal ion and phenyl ring of neotame at position 2. The MIA value at position 2 is high in comparison to position 1. The Mg^2+^-O bond length is 1.9 Å. Ca^2+^ ion is bicoordinated to O atom and N atom at position 1 whereas at positions 2 and 3 it is coordinated to O (5) and phenyl ring. MIA values show that Ca^2+^ ion is strongly interacting at position 3 (−159.64 kcal mol^−1^). The bond length between Ca^2+^-O is ~2.2 Å. In the case of metalation with Fe^2+^, the MIA is almost same at positions 2 and 3 with values of −238.45 kcal mol^−1^ and −239.71 kcal mol^−1^, respectively. The Fe^2+^-O bond length is ~1.9 Å in all the positions. Zn^2+^ ion is strongly coordinated with neotame at position 2 with MIA value of −223.39 kcal mol^−1^. There is a change in the configuration of the complex after optimization. The bond length between Zn^2+^ and O atom is same in all the positions (~2.0 Å). It is observed that in the case of divalent ions, there is a strong participation of aromatic ring in metal ion-neotame binding.

The MIA values for Li^+^, Na^+^ and K^+^ ions has been found to be −79.07 kcal mol^−1^, −56.16 kcal mol^−1^ and -45.18 kcal mol^−1^ at position 3 of neotame. It can be seen from Fig. 7 that at position 3, Li^+^ and K^+^ is bicoordinated whereas in the case of Na^+^ ion benzene ring of neotame is oriented in such a manner that it is also complexing with Na^+^ ion thereby forming a stable complex.

#### Metal ion complexes of α-fructose, β-fructose and neotame in water as solvent

The interaction of monovalent and divalent ions with α-fructose, β-fructose and neotame has been studied in water as solvent using B3LYP/6-311 + G (d, p) and B3LYP/D3 basis sets. The complexation of α-fructose, β-fructose and neotame in water using B3LYP/6-311-G (d, p) basis set has been shown in Figs. S1–S3 whereas complexation of α-fructose, β-fructose and neotame in water as solvent using B3LYP/D3 basis set has been depicted in Figs. S4–S6 respectively (see Supplementary Information).

#### Metal ion affinities of α, β-fructose and neotame with mono and divalent cations

The metal ion affinities of mono and divalent cations were calculated using B3LYP/6-311 + G (d, p) and B3LYP/D3 basis sets in gas phase.

Gas phase with B3LYP/6-311 + G (d, p) basis set: In the case of α-fructose, Li + ion monocoordinated at position 1, Na^+^ ion bicoordinated at positions 1, 3 and K^+^ ion at position 3 with energy values of −79.69 kcal mol^−1^, −52.08 kcal mol^−1^, −52.08 kcal mol^−1^ and −38.59 kcal mol^−1^ respectively. The β-fructose coordinated at positions 1, 3 with Li^+^ ion, position 3 with Na^+^ ion and position 3 with K^+^ ion having energy values of −80.32 kcal mol^−1^, −80.32 kcal mol^−1^, −48.95 kcal mol^−1^ and −16.94 kcal mol^−1^. Furthermore, neotame monocoordinated with Li^+^, Na^+^ and K^+^ ions at positions 3, 2 and 3 with energy values of −77.81 kcal mol^−1^, −147.69 kcal mol^−1^ and −42.04 kcal mol^−1^ respectively as shown in Table 1.

**Table 1:** Metal ion affinities (MIA) of α, β-fructose and neotame with monovalent cations using B3LYP/6-311 + G (d, p) basis set in gas phase.

Molecule	Li^+^ ion (kcal mol^−1^)			Na^+^ ion (kcal mol^−1^)			K^+^ ion (kcal mol^−1^)		
	Position 1	Position 2	Position 3	Position 1	Position 2	Position 3	Position 1	Position 2	Position 3
α- fructose	−63.38	−67.77	−79.69	−52.08	−38.28	−52.08	−36.39	−26.98	−38.59
β-fructose	−80.32	−68.39	−80.32	−45.93	−47.69	−48.95	−9.04	−12.55	−16.94
Neotame	−49.57	−66.52	−77.81	−53.34	−147.69	−52.08	−38.91	−34.51	−42.04

The energy values of Mg^2+^-α, β-fructose and neotame complexes were found to be −207.71 k calmol^−1^, −207.71 kcal mol^−1^, −231.55 kcal mol^−1^, −231.55 kcal mol^−1^ and −229.04 kcal mol^−1^ which has led to the bicoordination with α and β-fructose at positions 1, 3 and 2, 3. Moreover, Mg^2+^ monocoordinated with neotame at position 1. The Ca^2+^ ion monocoordinated with α, β-fructose and neotame at positions 1, 2 and 1 with energy values of −148.09 kcal mol^−1^, −166.92 kcal mol^−1^ and −148.09 kcal mol^−1^, respectively. The α, β-fructose and neotame monocoordinated with Fe^2+^ and Zn^2+^ ions at positions 3, 3 and 3 with metal ion affinities of −218.37 kcal mol^−1^, −246.61 kcal mol^−1^, −239.71 kcal mol^−1^ and −225.90 kcal mol^−1^, −247.87 kcal mol^−1^, −223.39 kcal mol^−1^ as represented in Table 2.

**Table 2:** Metal ion affinities (MIA) of α, β-fructose and neotame with divalent cations using B3LYP/6-311 + G (d, p) basis set in gas phase.

Divalent ions	α-fructose			β-fructose			Neotame		
	Position 1	Position 2	Position 3	Position 1	Position 2	Position 3	Position 1	Position 2	Position 3
Mg^2+^ (kcal mol^−1^)	−207.71	−163.15	−207.71	−200.18	−231.55	−231.55	−229.04	−200.18	−168.17
Ca^2+^ (kcal mol^−1^)	−148.09	−111.51	−166.29	−141.19	−166.92	−133.03	−148.09	−1 +++ 6.21	−159.64
Fe^2+^ (kcal mol^−1^)	−203.31	−202.69	−218.37	−195.16	−203.94	−246.61	−196.41	−238.45	−239.71
Zn^2+^ (kcal mol^−1^)	−212.09	−175.08	−225.90	−212.73	−219.63	−247.87	−192.02	−223.39	−203.94

Gas phase with B3LYP/D3 basis set: In the case of α-fructose, Li+, Na^+^ and K^+^ ions monocoordinated at positions 3, 1 and 1 with energy values of −179.47 kcal mol^−1^, −136.17 kcal mol^−1^ and −135.54 kcal mol^−1^ respectively. The β-fructose coordinated at positions 3, 3 and 3 with Li^+^, Na^+^ and K^+^ ions having energy values of −69.65 kcal mol^−1^, −50.95 kcal mol^−1^ and −38.91 kcal mol^−1^. Furthermore, neotame also monocoordinated with Li^+^, Na^+^ and K^+^ ions at positions 3, 3 and 3 with energy values of −79.07 kcal mol^−1^, −56.16 kcal mol^−1^ and −45.18 kcal mol^−1^, respectively as shown in Table 3.

**Table 3:** Metal ion affinities (MIA) of α, β-fructose and neotame with monovalent cations using B3LYP/D3 basis set in gas phase.

Molecule	Li^+^ ion (kcal mol^−1^)			Na^+^ ion (kcal mol^−1^)			K^+^ ion (kcal mol^−1^)		
	Position 1	Position 2	Position 3	Position 1	Position 2	Position 3	Position 1	Position 2	Position 3
α- fructose	−146.21	−142.45	−179.47	−136.17	−132.41	−133.72	−135.54	−110.44	−112.32
β- fructose	−60.49	−47.49	−69.65	−49.57	−49.57	−50.95	−36.21	−34.70	−38.91
Neotame	−76.24	−65.26	−79.07	−55.22	−49.69	−56.16	−41.42	−38.28	−45.18

The energy values of Mg^2+^-α, β-fructose and neotame complexes were found to be −210.84 kcal mol^−1^, −210.84 kcal mol^−1^, −202.67 kcal mol^−1^ and −215.42 kcal mol^−1^ which has led to the bicoordination with α-fructose at positions 1, 3 and monocoordinated with β-fructose and neotame at positions 1 and 3, respectively. The Ca^2+^ ion monocoordinated with α, β-fructose and neotame at positions 3, 2 and 3 with energy values of −150.92 kcal mol^−1^, −166.68 kcal mol^−1^ and −181.35 kcal mol^−1^, respectively. The Fe^2+^ and Zn^2+^ ions bicoordinated with α-fructose at positions 1, 3 and 1, 3 with metal ion affinities of −226.53 kcal mol^−1^, −226.53 kcal mol^−1^ kcal mol^−1^ and −229.67 kcal mol^−1^, −229.67 kcal mol^−1^, respectively. However, Fe^2+^ and Zn^2+^ ions monocoordinated with β-fructose and neotame at positions 1, 2 and 3, 2 with energy values of −260.28 kcal mol^−1^, −252.56 kcal mol^−1^ and −240.07 kcal mol^−1^, −261.34 kcal mol^−1^ as represented in Table 4.

**Table 4:** Metal ion affinities (MIA) of α, β-fructose and neotame with divalent cations using B3LYP/D3 basis set in gas phase.

Divalent ions	α-fructose			β-fructose			Neotame		
	Position 1	Position 2	Position 3	Position 1	Position 2	Position 3	Position 1	Position 2	Position 3
Mg (kcal mol^−1^)	−210.84	−158.76	−210.84	−202.67	−167.55	−186.37	−210.84	−202.69	−215.42
Ca (kcal mol^−1^)	−150.85	−114.21	−150.92	−130.27	−169.68	−136.54	−151.42	−149.35	−181.35
Fe (kcal mol^−1^)	−226.53	−180.09	−226.53	−260.28	−220.26	−208.08	−206.84	−210.30	−240.07
Zn (kcal mol^−1^)	−229.67	−179.47	−229.67	−252.26	−252.56	−207.71	−232.49	−261.34	−208.91

Interestingly, it was found that the metal ion affinities calculated with B3LYP/D3 basis set were much lower compared to B3LYP/6-311 + G (d, p) basis set. Overall metal ion affinities indicated that neotame bounded strongly Na^+^ ion followed by Li^+^ and K^+^ ions. Whereas in the case of divalent ions, Fe^2+^ ion strongly bounded with β-fructose followed by neotame and α-fructose calculated using B3LYP/6-311 + G (d, p) basis set. The α-fructose bounded strongly with Li^+^ followed by Na^+^ and K^+^ ions. On the other hand, neotame also exhibited similar trend. In the case of metal ion affinities calculated with B3LYP/D3 basis set, Zn^2+^ ion bounded strongly with neotame followed by Fe^2+^, Mg^2+^ and Ca^2+^ ions.

Water medium with B3LYP/6-311 + G (d, p) basis set: In the case of α-fructose, Li^+^ ion bicoordinated at positions 1, 3, whereas Na^+^ ion tricoordinated at positions 1, 2, 3 and K^+^ ion bicoordinated at positions1, 2 with energy values of −124.87 kcal mol^−1^, −124.87 kcal mol^−1^, −102.28 kcal mol^−1^, −102.28 kcal mol^−1^, −102.28 kcal mol^−1^ and −80.32 kcal mol^−1^, −80.32 kcal mol^−1^, respectively. The β-fructose coordinated at position 2 with Li^+^ ion, position 3 with Na^+^ ion and bicoordinated positions 2, 3 with K^+^ ion having energy values of −129.27 kcal mol^−1^, −106.05 kcal mol^−1^ and −83.46, −83.46 kcal mol^−1^, respectively. Furthermore, neotame monocoordinated with Li^+^, Na^+^ and K^+^ ions at positions 3, 1 and 3 with energy values of −127.38 kcal mol^−1^, −103.54 kcal mol^−1^ and −83.46 kcal mol^−1^, respectively as shown in Table 5.

**Table 5:** Metal ion affinities (MIA) of α-fructose, β-fructose and neotame with monovalent cations using B3LYP/6-311 + G (d, p) basis set (water).

Molecule	Li^+^ ion (kcal mol^−1^)			Na^+^ ion (kcal mol^−1^)			K^+^ ion (kcal mol^−1^)		
	Position 1	Position 2	Position 3	Position 1	Position 2	Position 3	Position 1	Position 2	Position 3
α-fructose	−124.87	−124.25	−124.87	−102.28	−102.28	−102.28	−80.32	−80.32	−80.26
β-fructose	−123.62	−129.27	−124.87	−101.66	−101.03	−106.05	−79.07	−83.46	−83.46
Neotame	−124.87	−124.87	−127.38	−103.54	−100.40	−100.40	−81.58	−82.20	−83.46

The energy values of Mg^2+^-α and β-fructose complexes were found to be −411.02 kcal mol^−1^, −410.39 kcal mol^−1^ and −410.39 kcal mol^−1^ which has led to the mono and bicoordination of α and β-fructose at positions 1 and 1, 2. Similarly, Mg^2+^-neotame was monocoordinated at position 3 with an metal ion affinity of −412.63 kcal mol^−1^. The Ca^2+^ ion monocoordinated with α, β-fructose and neotame at positions 1, 3 and 3 with energy values of −363.96 kcal mol^−1^, −362.70 kcal mol^−1^ and −362.07 kcal mol^−1^, respectively. The α, β-fructose and neotame monocoordinated with Fe^2+^ ion at positions 2, 2 and 2 with metal ion affinities of −426.08 kcal mol^−1^, −433.94 kcal mol^−1^, −424.82 kcal mol^−1^ respectively. Whereas, Zn^2+^ ion monocoordinated with α-fructose at position 1 with energy value of −440.51 kcal mol^−1^, however β-fructose and neotame were bicoordinated at positions 2, 3 and 1, 2 with metal ions affinities of −444.90 kcal mol^−1^, −444.90 kcal mol^−1^ and −439.88 kcal mol^−1^, −439.88 kcal mol^−1^ as represented in Table 6.

**Table 6:** Metal ion affinities (MIA) of α-fructose, β-fructose and neotame with divalent cations using B3LYP/6-311 + G (d, p) basis set (water).

Divalent ions	α-fructose			β-fructose			Neotame		
	Position 1	Position 2	Position 3	Position 1	Position 2	Position 3	Position 1	Position 2	Position 3
Mg^2+^ (kcal mol^−1^)	−411.02	−405.99	−403.49	−410.39	−410.39	−407.88	−405.99	−406.63	−412.63
Ca^2+^ (kcal mol^−1^)	−363.96	−355.79	−355.79	−353.29	−358.94	−362.70	−357.05	−358.31	−362.07
Fe^2+^ (kcal mol^−1^)	−421.69	−426.08	−421.69	−422.94	−433.94	−428.59	−423.57	−424.82	−414.78
Zn^2+^ (kcal mol^−1^)	−440.51	−441.14	−438.63	−439.88	−444.90	−444.90	−439.88	−439.88	−432.35

Water medium with B3LYP/D3 basis set: In the case of α-fructose, Li^+^ and K^+^ ions were monocoordinated at positions 3 and 1 with energy values of −144.96 kcal mol^−1^and −102.91 kcal mol^−1^, respectively. Whereas, Na^+^ ion bicoorindated at positions 1 and 3 with energy values of −124.12 kcal mol^−1^ and −124.12 kcal mol^−1^. The β-fructose coordinated at positions 1, 2, 3 and 3 with Li^+^, Na^+^ and K^+^ ions having energy values of −128.01 kcal mol^−1^, −106.05 kcal mol^−1^, −106.05 kcal mol^−1^ and −88.48 kcal mol^−1^. Furthermore, neotame also monocoordinated with Li^+^, Na^+^ and K^+^ ions at positions 3, 1 and 3 with energy values of −65.26 kcal mol^−1^, −41.16 kcal mol^−1^ and −21.02 kcal mol^−1^, respectively as shown in Table 7.

**Table 7:** Metal ion affinities (MIA) of α-fructose, β-fructose and neotame with monovalent cations using B3LYP/D3 basis set (water).

Molecule	Li^+^ ion (kcal mol^−1^)			Na^+^ ion (kcal mol^−1^)			K^+^ ion (kcal mol^−1^)		
	Position 1	Position 2	Position 3	Position 1	Position 2	Position 3	Position 1	Position 2	Position 3
α-fructose	−143.07	−141.19	−144.96	−124.12	−121.36	−124.12	−102.91	−99.15	−101.66
β-fructose	−128.01	−126.13	−126.76	−104.79	−106.05	−106.05	−82.83	−84.09	−88.48
Neotame	−62.12	−63.38	−65.26	−41.16	−38.28	−38.47	−19.89	−19.52	−21.02

The energy values of Mg^2+^-α, β-fructose and neotame complexes were found to be −429.84 kcal mol^−1^, −420.43 kcal mol^−1^ and −394.08 kcal mol^−1^ which has led to the monocoordination at positions 3, 3 and 3, respectively. The Ca^2+^ ion monocoordinated with α, β-fructose and neotame at positions 1, 3 and 3 with energy values of −375.88 kcal mol^−1^, −367.72 kcal mol^−1^ and −343.88 kcal mol^−1^, respectively. The Fe^2+^ and Zn^2+^ ions monocoordinated with α-fructose at positions 3 and 3 with metal ion affinities of −448.76 kcal mol^−1^ and −470.63 kcal mol^−1^ respectively. Similarly, Fe^2+^ and Zn^2+^ ions monocoordinated with β-fructose and neotame at positions 3, 2 and 3, 3 with energy values of −439.88 kcal mol^−1^, −447.41 kcal mol^−1^ and −436.32 kcal mol^−1^, −448.89 kcal mol^−1^ as represented in Table 8.

**Table 8:** Metal ion affinities (MIA) of α-fructose, β-fructose and neotame with divalent cations using B3LYP/D3 basis set (water).

Divalent ions	α-fructose			β-fructose			Neotame		
	Position 1	Position 2	Position 3	Position 1	Position 2	Position 3	Position 1	Position 2	Position 3
Mg^2+^ (kcal mol^−1^)	−426.08	−426.68	−429.84	−408.51	−411.02	−420.43	−387.17	−380.27	−394.08
Ca^2+^ (kcal mol^−1^)	−375.88	−374.62	−376.33	−358.31	−361.45	−367.72	−340.11	−341.62	−343.88
Fe^2+^ (kcal mol^−1^)	−444.28	−444.90	−448.76	−427.96	−426.08	−439.88	−426.10	−427.90	−436.32
Zn^2+^ (kcal mol^−1^)	−463.10	−462.16	−470.63	−444.90	−447.41	−446.16	−445.24	−446.47	−448.89

Almost similar trend has been observed in the case of metal ion affinities calculated in water medium using both B3LYP/D3 and B3LYP/6-311 + G (d, p) basis sets.

#### Comparison of ∆G values of α, β-fructose and neotame with mono and divalent cations

Gas phase with B3LYP/6-311 + G (d, p) basis set: The Gibbs Energy (**∆**G) values were calculated for α, β-fructose and neotame with mono and divalent cations using B3LYP/6-311 + G (d, p) and B3LYP/D3 basis set in gas phase and water medium. The obtained results were presented in Tables 9–16. It was found that the metalation of Li^+^, Na^+^ and K^+^ ions appears to be bicoordinated (**∆**G = −64.88 kcal mol^−1^ for both 1 and 3 positions), monocoordinated (−42.86 kcal mol^−1^ at position 1 and −29.62 kcal mol^−1^ at position 3) with α-fructose respectively in gas phase. The **∆**G values for Li^+^, Na^+^ and K^+^ ions with β-fructose and neotame coordinated at position 1 and 3 with ∆G values of −68.34 kcal mol^−1^, −45.12 kcal mol^−1^ and −33.89 kcal mol^−1^, respectively. In the case of monovalent ions, neotame binds strongly with Li^+^ ion compared to Na^+^ and K^+^ ions as shown in Table 9.

**Table 9:** ∆G values of α, β-fructose and neotame with monovalent cations B3LYP/6-311 + G(d,p) basis set in gas phase.

Molecule	Li^+^ ion (kcal mol^−1^)			Na^+^ ion (kcal mol^−1^)			K^+^ ion (kcal mol^−1^)		
	Position 1	Position 2	Position 3	Position 1	Position 2	Position 3	Position 1	Position 2	Position 3
α-fructose	−64.88	−45.43	−64.88	−42.86	−30.18	−42.82	−28.36	−19.59	−29.62
β- fructose	−71.54	−59.43	−71.22	−37.21	−38.94	−40.09	−18.64	−23.85	−27.61
Neotame	−39.97	−56.85	−68.34	−45.12	−38.22	−42.92	−31.06	−28.18	−33.89

**Table 10:** ∆G values of α, β-fructose and neotame with divalent cations B3LYP/6-311 + G (d, p) basis set in gas phase.

Divalent ions	α-fructose			β-fructose			Neotame		
	Position 1	Position 2	Position 3	Position 1	Position 2	Position 3	Position 1	Position 2	Position 3
Mg^2+^ (kcal mol^−1^)	−196.79	−151.86	−196.79	−189.51	−220.26	−220.26	−217.99	−190.07	−159.39
Ca^2+^ (kcal mol^−1^)	−137.80	−102.59	−154.87	−131.34	−156.25	−123.12	−137.61	−136.67	−149.35
Fe^2+^ (kcal mol^−1^)	−194.97	−−192.02	−207.71	−187.56	−193.39	−234.94	−188.82	−229.61	−229.67
Zn^2+^ (kcal mol^−1^)	−200.89	−166.35	−215.24	−202.06	−209.09	−236.57	−186.37	−214.11	−195.53

**Table 11:** ∆G values of α, β-fructose and neotame with monovalent cations using B3LYP/D3 basis set in gas phase.

Molecule	Li^+^ ion (kcal mol^−1^)			Na^+^ ion (kcal mol^−1^)			K^+^ ion (kcal mol^−1^)		
	Position 1	Position 2	Position 3	Position 1	Position 2	Position 3	Position 1	Position 2	Position 3
α- fructose	−138.18	−136.19	−171.49	−128.89	−126.63	−126.88	−129.14	−104.79	−105.55
β- fructose	−50.95	−40.16	−60.87	−41.79	−40.39	−40.79	−26.79	−25.60	−28.87
Neotame	−67.33	−55.66	−69.15	−46.44	−41.42	−46.81	−33.57	−30.49	−36.33

**Table 12:** ∆G values of α, β-fructose and neotame with divalent cations using B3LYP/D3 basis set in gas phase.

Divalent ions	α-fructose			β-fructose			Neotame		
	Position 1	Position 2	Position 3	Position 1	Position 2	Position 3	Position 1	Position 2	Position 3
Mg^2+^ (kcal mol^−1^)	−199.74	−149.15	−199.74	−191.39	−154.99	−175.08	−200.30	−189.38	−205.76
Ca^2+^ (kcal mol^−1^)	−140.62	−105.29	−140.62	−118.72	−157.69	−125.31	−141.94	−139.31	−170.06
Fe^2+^ (kcal mol^−1^)	−215.86	−170.31	−215.93	−188.88	−208.96	−196.41	−189.76	−209.96	−197.40
Zn^2+^ (kcal mol^−1^)	−219.00	−170.06	−218.94	−239.71	−239.71	−196.41	−238.70	−240.82	−195.48

**Table 13:** ∆G values of α, β-fructose and neotame with monovalent cations B3LYP/6-311 + G (d, p) basis set in water medium.

Molecule	Li ion (kcal mol^−1^)			Na ion (kcal mol^−1^)			K ion (kcal mol^−1^)		
	Position 1	Position 2	Position 3	Position 1	Position 2	Position 3	Position 1	Position 2	Position 3
α- fructose	−116.09	−118.59	−116.09	−94.75	−96.64	−94.64	−74.05	−74.67	−74.05
β- fructose	−114.83	−120.48	−116.72	−93.49	−93.49	−98.52	−72.16	−75.30	−75.56
Neotame	−116.72	−116.72	−119.23	−95.38	−94.75	−94.75	−74.67	−74.67	−77.18

**Table 14:** ∆G values of α, β-fructose and neotame with divalent cations B3LYP/6-311 + G (d, p) basis set in water medium.

Divalent ions	α-fructose			β-fructose			Neotame		
	Position 1	Position 2	Position 3	Position 1	Position 2	Position 3	Position 1	Position 2	Position 3
Mg^2+^ (kcal mol^−1^)	−400.98	−400.35	−393.45	−333.84	−400.98	−399.72	−396.59	−395.96	−404.74
Ca^2+^ (kcal mol^−1^)	−354.54	−349.52	−347.64	−343.86	−350.79	−353.92	−348.89	−348.89	−353.29
Fe^2+^ (kcal mol^−1^)	−412.27	−417.92	−416.75	−413.53	−424.19	−419.18	−414.78	−413.53	−404.74
Zn^2+^ (kcal mol^−1^)	−442.39	−444.90	−441.14	−429.84	−435.49	−432.35	−430.47	−431.09	−404.74

**Table 15:** ∆G values of α, β-fructose and neotame with monovalent cations using B3LYP/D3 basis set in water medium.

Molecule	Li^+^ ion (kcal mol^−1^)			Na^+^ ion (kcal mol^−1^)			K^+^ ion (kcal mol^−1^)		
	Position 1	Position 2	Position 3	Position 1	Position 2	Position 3	Position 1	Position 2	Position 3
α- fructose	−118.59	−117.97	−119.85	−99.77	−98.52	−99.77	−79.69	−76.56	77.18
β-fructose	−119.85	−118.59	−119.85	−97.26	−99.15	−99.15	−75.30	−77.18	−80.32
Neotame	−117.34	−119.97	−119.85	−99.15	−98.52	−98.52	−76.56	−77.18	−77.18

**Table 16:** ∆G values of α-fructose, β-fructose and neotame with divalent cations using B3LYP/D3 basis set in water medium.

Divalent ions	α-fructose			β-fructose			Neotame		
	Position 1	Position 2	Position 3	Position 1	Position 2	Position 3	Position 1	Position 2	Position 3
Mg^2+^ (kcal mol^−1^)	−400.35	−400.98	−404.12	−398.47	−402.86	−412.27	−398.47	−391.57	−407.88
Ca^2+^ (kcal mol^−1^)	−350.15	−350.78	−352.03	−349.52	−353.92	−358.31	−350.78	−353.29	−355.79
Fe^2+^ (kcal mol^−1^)	−426.71	−420.43	−423.57	−419.18	−370.86	−429.22	−415.80	−378.70	−412.98
Zn^2+^ (kcal mol^−1^)	−437.37	−436.75	−443.65	−436.12	−438.00	−437.34	−437.10	−439.18	−438.30

In the case of Mg^2+^, α and β-fructose appears to be bicoordinated at positions 1, 3 and 2, 3, whereas neotame is monocoordinated at position 1 with **∆**G values of −196.79 kcal mol^−1^, −189.51 kcal mol^−1^ and −217.99 kcal mol^−1^, respectively. The Ca^2+^ ion chelates with α, β-fructose and neotame at positions 4, 3, and 3 with **∆**G values of −154.87 kcal mol^−1^, −156.25 kcal mol^−1^ and −149.35 kcal mol^−1^. The Fe^2+^ ions monocoordinated with α, β-fructose and neotame at positions 3, 3, and 2 with ∆G values of −207.71 kcal mol^−1^, −234.94 kcal mol^−1^, −229.67 kcal mol^−1^. The **∆**G values for Zn^2+^ coordinated at position 3 with α, β-fructose and at position 2 with neotame were found to be −215.24, −236.57 and −214.11 kcal mol^−1^ respectively. The obtained results reveal that Zn^2+^ bounded strongly with β-fructose followed by α-fructose and neotame as represented in Table 10.

Gas phase with B3LYP/D3 basis set: The Li^+^, Na^+^ and K^+^ ions coordinates at positions 3, 1, 1 with α-fructose with **∆**G values of −171.49 kcal mol^−1^, −128.89 kcal mol^−1^, −129.14 kcal mol^−1^, respectively. In the case of β-fructose and neotame, the coordination is at single positions 3, 1, 2 and 3, 3, 3, respectively. The **∆**G values for β-fructose and neotame complexes were found to be −60.87 kcal mol^−1^, −41.79 kcal mol^−1^, 28.87 kcal mol^−1^ and −69.15 kcal mol^−1^, −46.81 kcal mol^−1^, −36.33 kcal mol^−1^, respectively as shown in Table 11.

The Mg^2+^ ion coordinated with α and β-fructose strongly at positions 1, 3 and 1 with **∆**G values of −199.74 kcal mol^−1^, −199.74 kcal mol^−1^ and −191.39 kcal mol^−1^. Whereas in the case of neotame, Mg^2+^ ion coordinated at position 3 with **∆**G value of −205.76 kcal mol^−1^. The Ca^2+^ ions bicoordinated with α, β-fructose and monocoordinated with neotame at positions 1, 3, 3 and 3 respectively. The **∆**G values of Ca^2+^- α, β-fructose and neotame complexes were found to be −140.62 kcal mol^−1^, −140.62 kcal mol^−1^, −125.31 kcal mol^−1^ and −170.06 kcal mol^−1^ respectively. Likewise in monovalent ions, Fe^2+^ ions also monocoordinated with α, β-fructose and neotame at positions 1, 2 and 2 having **∆**G value of −215.93 kcal mol^−1^, −208.96 kcal mol^−1^, −209.96 kcal mol^−1^, respectively. The Zn^2+^ ions appear to be monocoordinated with α-fructose and bicoordinated with β-fructose at positions 1 and 1, 2 with ∆G values of –219.00 kcal mol^−1^, −239.71kcal mol^−1^ and −239.71 kcal mol^−1^. In the case of Zn^2+^-neotame complex, ∆G is found to be −240.80 kcal mol^−1^ with a monocoordinated at position 2. After scrutiny of the obtained results, Zn^2+^ ion bounded strongly with neotame as compared to α, β-fructose as shown in Table 12.

Water medium with B3LYP/6-311 + G (d, p) basis set: It was found that the metalation of Li^+^, Na^+^ and K^+^ ions appears to be monocoordinated at position 2 with α-fructose and ∆G values were found to be −118.59 kcalmol^−1^, −96.64 kcalmol^−1^, −74.67 kcal mol^−1^. The ∆G values for Li^+^, Na^+^ and K^+^ ions with β-fructose and neotame coordinated at positions 2, 3, 3 with values of −120.48 kcalmol^−1^, −98.52 kcal mol^−1^ and −75.56 kcal mol^−1^, whereas neotoame also monocoordinated with Li^+^, Na^+^ and K^+^ ions at positions 3, 1 and 3. The ∆G values were recorded as −119.23 kcal mol^−1^, −95.38 kcal mol^−1^ and −77.18 kcal mol^−1^, respectively as shown in Table 13. The obtained results suggested that Li^+^ ions strongly bounded with neotame.

In the case of Mg^2+^, α and β-fructose appears to be monocoordinated at positions 1 and 2, whereas neotame is also monocoordinated at position 3 with ∆G values of −400.98 kcal mol^−1^, −400.98 kcal mol^−1^ and −404.74 kcalmol^−1^, respectively. The Ca^2+^ ion chelates with α, β-fructose and neotame at positions 1, 3, and 3 with ∆G values of −354.54 kcal mol^−1^, −353.92 kcal mol^−1^ and −353.29 kcal mol^−1^. The Fe^2+^ ions monocoordinated with α, β-fructose and neotame at positions 2, 2, and 1 with ∆G values of −417.92 kcal mol^−1^, −424.19 kcal mol^−1^, −414.78 kcal mol^−1^. The ∆G values for Zn^2+^ monocoordinated as positions 2 with α, β-fructose and neotame were found to be −444.90 kcal mol^−1^, −435.49 kcal mol^−1^ and −431.09 kcal mol^−1^, respectively. The obtained results reveal that Zn^2+^ binds strongly with α-fructose followed by β-fructose and neotame as represented in Table 14.

Water medium with B3LYP/D3 basis set: The Li^+^, Na^+^ and K^+^ ions coordinates at positions 3, 1, 3 and 1 with α-fructose with ∆G values of −119.85 kcal mol^−1^, −99.77 kcal mol^−1^, −99.77 kcal mol^−1^ and −79.69 kcal mol^−1^, respectively. In the case of β-fructose, Li^+^ and Na^+^ ions bicoordinated at positions 1, 3 and 2, 3. Whereas, neotame monocoordinated with Li^+^, Na^+^ ions and bicoordinated with K^+^ ion at positions 3, 1 and 2, 3, respectively. The ∆G values for β-fructose and neotame complexes were found to be −119.85 kcalmol^−1^, −119.85 kcalmol^−1^, −99.15 kcal mol^−1^, −99.15 kcal mol^−1^, −80.32 kcal mol^−1^ and −119.85 kcal mol^−1^, −99.15 kcal mol^−1^, −77.18 kcal mol^−1^, −77.18 kcal mol^−1^ respectively as shown in Table 15. The obtained results suggested that Li ^+^ ions strongly bounded with neotame compared to Na^+^ and K^+^ ions.

The Mg^2+^ ion monocoordinated with α and β-fructose strongly at positions 3, 3, 3 and 3, 3, 3 with ∆G values of −404.12 kcal mol^−1^, −412.27 kcal mol^−1^ and −352.03 kcal mol^−1^, −358.31 kcal mol^−1^, respectively. Whereas in the case of neotame, Mg^2+^ ion coordinated at position 3 with ∆G value of −407.88 kcal mol^−1^. The Ca^2+^ ions monocoordinated with α, β-fructose and neotame at positions 3, 3 and 3, respectively. The ∆G values of Ca^2+^- α, β-fructose and neotame complexes were found to be −352.03 kcal mol^−1^, −358.31 kcal mol^−1^ and −355.79 kcal mol^−1^, respectively. Like wise in monovalent ions, Fe^2+^ ions also monocoordinated with α, β-fructose and neotame at positions 1, 3 and 1 having ∆G value of −426.71 kcal mol^−1^, −429.22 kcal mol^−1^, −415.80 kcal mol^−1^, respectively. The Zn^2+^ ions appear to be monocoordinated with α, β-fructose at positions 3 and 2 with ∆G values of –443.65 kcal mol^−1^ and −438.00 kcal mol^−1^. In the case of Zn^2+^-neotame complex, ∆G is found to be −439.18 kcal mol^−1^ with a monocoordinated at position 2. After scrutiny of the obtained results, Zn^2+^ ion binds strongly with neotame as compared to α, β-fructose as shown in Table 16.

Overall results indicated that the calculated free energy (ΔG) values using B3LYP/6-311 + G(d,p) basis set in gas phase showed the strong binding of Li^+^ ion with β-fructose compared to α-fructose and neotame. The same trend in the ΔG values were observed in water medium for monovalent ions. Whereas in the case of divalent ions, neotame interacts strongly with Zn^2+^ ion compared to Fe^2+^, Ca^2+^ and Mg^2+^ ions using B3LYP/D3 basis set in gas phase. The same trend was observed with ΔG values in water medium.

### Experimental discussion

#### Characterization of neotame-Zn^2+^ complex

The fourier-transform infrared (FTIR) and UV-visible spectroscopy techniques were used to characterize the interaction of neotame with Zn^2+^ ions.

Fourier transform infrared spectroscopy (FTIR): The neotame-Zn^2+^ was analyzed using FT-IR. In neotame, predominant absorption peaks were observed at 1598 cm^−1^(*s*), 1691 cm^−1^(*s*) and 1729 cm^−1^(*s*) which have been attributed to the carboxyl carbonyl (-C = O), ester carbonyl (-C = O) and amide carbonyl (-C = O) groups, respectively in zwitterion form. A small sharp peak at 1544 cm^−1^(*b*) corresponds to the β-amino group (-NH) of neotame. In the case of neotame-Zn^2+^ complex, β-amino group (-NH) (1598 cm^−1^) and carboxylic carbonyl (-C = O) (1544 cm^−1^) were shifted to 1568 cm^−1^ and 1627 cm^−1^, respectively which indicates that β-amino group (-NH) and carboxyl carbonyl (-C = O) groups were involved in the chelation process. It was found that the experimental results were exactly corresponding to the theoretical results. The FTIR spectrum for neotame and neotame-Zn^2+^ complex recorded experimentally and theoretically was shown in Fig. S7 (see Supplementary Information).

UV-visible spectroscopy: The UV-Visible spectroscopy was used to confirm the formation of neotame-Zn^2+^ complex, stoichiometry and its stability constant. Interestingly, it was found that neotame-Zn^2+^ shows two absorption bands in the UV-visible region. The absorption band-1 of the complex corresponds to the neotame-Zn^2+^ charge transfer band, while their absorbance vs the molar ratios of neotame to Zn^2+^ was obtained by varying the neotame concentration, resulting with a molar ratio of 1.0 as shown in Fig. S8 (see Supplementary Information). The obtained data suggest that one molecule of neotame interacts with the Zn^2+^ ion resulting in neotame-Zn^2+^ complex. The stoichiometry ratio of the complex was found to be 1:1 (Neotame: Zn^2+^) and confirmed by the Job’s method as well as with a literature report^5^. The Harvey and Manning equation^30^ was used to calculate the conditional formation constant (log K) of neotame-Zn^2+^ complex and found to be 4.09.

#### Electrochemical evaluation of neotame-Zn^2+^ complex

**Figure 8:**
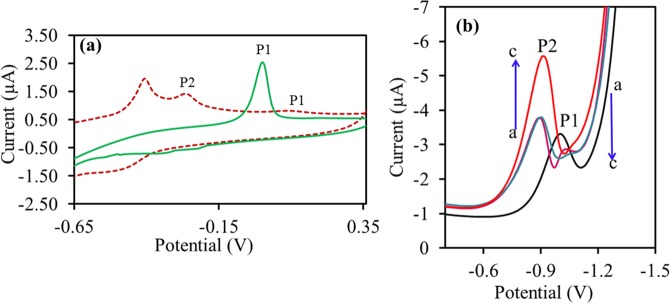
(**a**) Cyclic voltammogram for 1 mM ZnCl_2_ (green solid line) and 6 mM of neotame solution to the ZnCl_2_ (red dotted line) (**b**) Differential pulse voltammogram for ZnCl_2_ (P1) and neotame-Zn^2+^ complex (P2) with a scan rate of 0.11 v s^−1^ at pH 5.5.

The cyclic voltammogram of 2 mM of ZnCl_2_.2H_2_O exhibited one peak (P1) at −1.13 V due to the reduction of Zn^2+^ in 0.1 M phosphate buffer medium at pH 5.5. Whereas, addition of 6-fold higher concentration of neotame to the ZnCl_2_.2H_2_O resulted in a new irreversible cathodic peak at −0.83, due to the reduction of neotame-Zn^2+^ complex as shown in Fig. 8(a). The differential pulse voltammetric results agree with the cyclic voltammetry, in which a cathodic peak at −1.13 V was due to the reduction of ZnCl_2_.2H_2_O. Interestingly, it was found that addition of higher concentration of neotame to the ZnCl_2_.2H_2_O, resulted a slight decrease in the peak current at −0.11 V (P1) and whereas, the peak current of neotame-Zn^2+^ increases at −0.85 V (P2) with a slight shift in the potentials as shown in Fig. 8(b).

### Comparison of computational and electrochemical studies: Neotame-Zn^2+^ complex as a case study

In the present study coordination of Zn^2+^ with neotame complex has been considered for the comparison between the computational and experimental results. The DFT results predict that neotame coordinates strongly with Zn^2+^ and Fe^2+^ relative to other monovalent and divalent ions (Table 2). The electrochemical evaluation of neotame-Zn^2+^ was carried out using cyclic voltammetry which showed an effective charge transfer between neotame and Zn^2+^. Considering the strong coordination between the two, the computational and experimental results have been found be comparable. Therefore, the information from the computational study for other monovalent and divalent ions can be used for the experimental study of complexation and stereochemistry of metal ions with different saccharide molecules.

## Implication of the Computational and Experimental Results

The present work serves to gain an insight into the understanding of the interaction of α-, β-fructose and neotame with mono and divalent ions using B3LYP/6-311 + G(d,p), B3LYP/D3 basis sets in gas phase and water medium. The interaction of metal ions with sugar molecules occur in biological system and plays a vital role in many metabolic activities. Based on the MIA values, metal ion affinities indicated that neotame bounded strongly Na^+^ ion followed by Li^+^ and K^+^ ions. Whereas in the case of divalent ions, Fe^2+^ ion strongly bounded with β-fructose followed by neotame and α-fructose calculated using B3LYP/6-311 + G (d, p) basis set. The α-fructose bounded strongly with Li^+^ followed by Na^+^ and K^+^ ions. On the other hand, neotame also exhibited similar trend. In the case of metal ion affinities calculated with B3LYP/D3 basis set, Zn^2+^ ion bounded strongly with neotame followed by Fe^2+^, Mg^2+^ and Ca^2+^ ions. In terms of ΔG values obtained from this study using B3LYP/6-311 + G(d,p) basis set in gas phase showed the strong binding of Li^+^ ion with β-fructose compared to α-fructose and neotame. The same trend in the ΔG values were observed in water medium for monovalent ions. Whereas in the case of divalent ions, neotame interacts strongly with Zn^2+^ ion compared to Fe^2+^, Ca^2+^ and Mg^2+^ ions using B3LYP/D3 basis set in gas phase. The same trend was observed with ΔG values in water medium. Interestingly, lower ΔG values are observed for α, β-fructose and neotame-metal ion system in water medium compared to gas phase. It could be attributed due to the interaction of water molecules with the complexes.

The results suggest that sugar molecules bind with ions through hydroxyl groups and oxygen atoms present in sugar molecules thereby acting as suitable sites for metalation.

The DFT calculations have helped in the identification of a preferred position in the α-, β-fructose and neotame for the interaction of metal ions. The MIA values and bond distances helps to gain reliable information about the coordination behaviour and complexation of sugar molecules with metal ions which is hampered by the difficulties while preparing the crystal structures. The investigation and study of these interactions paves a way to understand the chemical or biological nature of sugar molecules along with the biochemical properties of artificial sweeteners.

## Conclusions

The present study suggests that neotame as artificial sweetener and α-, β-fructose as natural sugar have dissimilar intrinsic thermo-chemical features using B3LYP/6-311 + G(d,p), B3LYP/D3 basis sets in gas phase and water medium. The MIA results indicate the strong chelation of neotame with metal (both mono and divalent). Based on the results in Tables 1 and 2, it is confirmed that neotame has an ability to donate electrons to the metal ions due to the presence of β-amino group (-NH), carboxyl carbonyl (-C = O) groups. It was demonstrated experimentally using FTIR results, which was further supported with theoretical results.

To the best of our knowledge, this work has been undertaken for the first time to predict the possible binding sites of mono, divalent metal ions with neotame and α-, β-fructose using DFT, electrochemical and spectroscopic studies. The present approach for metal binding affinity offer a promising strategy for better understanding of specific metabolic activity in cell wall and the glucose transmembrane transport. Further work on the chelation of metal ions with neotame could lead to the design of novel electrochemical biosensor for the detection of wide range of metal ions in various matrices.

## Supplementary information

Supplementary Information
