# Supplementary material for: Modeling of neotame and fructose thermochemistry: Comparison with mono and divalent metal ions by Computational and experimental approach
Source: Sci Rep. 2019 Dec 5;9:18414. doi: 10.1038/s41598-019-54626-9 (PMC6895154; doi:10.1038/s41598-019-54626-9)
Supplement: Supplementary file 1 — Supplementary Information [file 41598_2019_54626_MOESM1_ESM.docx]

**Modeling of neotame and fructose thermochemistry: Comparison with mono and divalent metal ions by Computational and experimental approach**

Deepali Sharma^1^, Suvardhan Kanchi^2*^, Ayyappa Bathinapatla^2^, Inamuddin^3,4,5*^, Abdullah M. Asiri^3,4^

^1^Department of Pharmaceutical Sciences, University of KwaZulu-Natal, Durban 4000, South Africa

^2*^Department of Chemistry, Faculty of Applied Science, Durban University of Technology, Durban, 4000 South Africa

^3*^Chemistry Department, Faculty of Science, King Abdulaziz University, Jeddah 21589, Saudi Arabia

^4*^Centre of Excellence for Advanced Materials Research, King Abdulaziz University, Jeddah 21589, Saudi Arabia

^5^Advanced Functional Materials Laboratory, Department of Applied Chemistry, Faculty of Engineering and Technology, Aligarh Muslim University, Aligarh- 202 002, India

^*^Corresponding authors:

Suvardhan Kanchi ([ksuvardhan@gmail.com](mailto:ksuvardhan@gmail.com)), Deepali Sharma ([dpschem@gmail.com](mailto:dpschem@gmail.com)) and Inamuddin ([inamuddin@zhcet.ac.in](mailto:inamuddin@zhcet.ac.in)).

**Supplementary materials**

**Figure S1**. Complexation of α-fructose with mono and divalent ions at three positions in water medium using B3LYP/6-311+G (d, p) basis set.

**Figure S2**. Complexation of β-fructose with mono and divalent ions at three positions in water medium using B3LYP/6-311+G (d, p) basis set.

**Figure S3**. Complexation of neotame with mono and divalent ions at three positions in water using B3LYP/6-311+G (d, p) basis set.

**Figure S4**. Complexation of α-fructose with mono and divalent ions at three positions in water medium using B3LYP/D3 basis set.

**Figure S5**. Complexation of β-fructose with mono and divalent ions at three positions in water medium using B3LYP/D3 basis set.

**Figure S6**. Complexation of neotame with mono and divalent ions at three positions in water medium using B3LYP/D3 basis set.

**Figure S7**. FTIR spectra of (i) neotame (a), neotame-Zn^2+^ complex (b) recorded experimentally (ii) and (iii) neotame-Zn^2+^ complex recorded computationally in gas phase and water medium.

**Figure S8.** UV-Visible spectra of (a) neotame and neotame-Zn2+ complex (b) Mole ratio method confirming the complex formation as 1:1 [Neotame: Zn^2+^].

**Cartesian coordinates of Neotame-Zn^2+^ complex optimized using 6-311G (d, p) basis set in gas phase for position 1**

| O | 0.3043 | 2.00249 | -1.84652 |
| --- | --- | --- | --- |
| O | 1.31035 | -1.82051 | 1.06726 |
| O | -2.71516 | 5.11528 | 0.93723 |
| O | -3.97475 | 3.30867 | 0.50667 |
| O | 2.23854 | -2.28066 | -0.93564 |
| N | -2.29949 | 0.78834 | 0.34856 |
| N | 0.33722 | 0.47166 | -0.1545 |
| C | -4.25005 | -2.53774 | -0.18996 |
| C | -3.56517 | -1.32095 | 0.48411 |
| C | -2.88808 | -0.30808 | -0.44099 |
| C | -5.39403 | -2.08657 | -1.11889 |
| C | -4.83939 | -3.42081 | 0.92712 |
| C | -3.22983 | -3.36726 | -0.99308 |
| C | -1.67143 | 1.82395 | -0.47187 |
| C | -1.55939 | 3.15757 | 0.28304 |
| C | -0.2435 | 1.43568 | -0.91132 |
| C | 1.70363 | 0.02131 | -0.37849 |
| C | 2.71067 | 0.80812 | 0.50528 |
| C | -2.88451 | 3.82562 | 0.56777 |
| C | 4.15897 | 0.44787 | 0.26247 |
| C | 1.79364 | -1.48464 | -0.15123 |
| C | 4.87177 | -0.32899 | 1.18084 |
| C | 4.81424 | 0.88071 | -0.89692 |
| C | 6.20426 | -0.66887 | 0.94903 |
| C | 6.14386 | 0.54193 | -1.13262 |
| C | 6.84357 | -0.23551 | -0.20988 |
| C | 1.34522 | -3.22381 | 1.39656 |
| H | -4.32017 | -0.79255 | 1.0814 |
| H | -2.81596 | -1.68842 | 1.19512 |
| H | -2.08418 | -0.78704 | -1.00699 |
| H | -3.60686 | 0.08468 | -1.17546 |
| H | -5.02763 | -1.5068 | -1.96995 |
| H | -5.92684 | -2.95434 | -1.51979 |
| H | -6.12043 | -1.47004 | -0.57986 |
| H | -5.33868 | -4.29972 | 0.50797 |
| H | -5.57566 | -2.86917 | 1.52015 |
| H | -4.05735 | -3.77266 | 1.60748 |
| H | -2.81939 | -2.80803 | -1.83794 |
| H | -2.39374 | -3.68404 | -0.36109 |
| H | -3.70033 | -4.26894 | -1.39678 |
| H | -2.22322 | 2.00471 | -1.40609 |
| H | -3.04538 | 1.21684 | 0.88736 |
| H | -0.93791 | 3.85094 | -0.28694 |
| H | -1.05786 | 3.00688 | 1.24689 |
| H | 1.93634 | 0.19433 | -1.42848 |
| H | -0.21049 | 0.10868 | 0.61794 |
| H | 2.45097 | 0.64804 | 1.55485 |
| H | 2.54568 | 1.86611 | 0.28329 |
| H | 4.38204 | -0.66466 | 2.08969 |
| H | 4.27954 | 1.49112 | -1.61798 |
| H | 6.74184 | -1.26925 | 1.6747 |
| H | 6.63532 | 0.88695 | -2.03551 |
| H | -3.59265 | 5.47919 | 1.13126 |
| H | 7.87945 | -0.49811 | -0.3925 |
| H | 0.92099 | -3.29944 | 2.39522 |
| H | 2.37268 | -3.58894 | 1.38462 |
| H | 0.75287 | -3.79567 | 0.68128 |
| Zn | -1.976 | 6.89509 | 1.13654 |

**Cartesian coordinates of Neotame-Zn^2+^ complex optimized using 6-311G (d, p) basis set in gas phase for position 2**

| O | 0.3043 | 2.00249 | -1.84652 |
| --- | --- | --- | --- |
| O | 1.31035 | -1.82051 | 1.06726 |
| O | -2.71516 | 5.11528 | 0.93723 |
| O | -3.97475 | 3.30867 | 0.50667 |
| O | 2.23854 | -2.28066 | -0.93564 |
| N | -2.29949 | 0.78834 | 0.34856 |
| N | 0.33722 | 0.47166 | -0.1545 |
| C | -4.25005 | -2.53774 | -0.18996 |
| C | -3.56517 | -1.32095 | 0.48411 |
| C | -2.88808 | -0.30808 | -0.44099 |
| C | -5.39403 | -2.08657 | -1.11889 |
| C | -4.83939 | -3.42081 | 0.92712 |
| C | -3.22983 | -3.36726 | -0.99308 |
| C | -1.67143 | 1.82395 | -0.47187 |
| C | -1.55939 | 3.15757 | 0.28304 |
| C | -0.2435 | 1.43568 | -0.91132 |
| C | 1.70363 | 0.02131 | -0.37849 |
| C | 2.71067 | 0.80812 | 0.50528 |
| C | -2.88451 | 3.82562 | 0.56777 |
| C | 4.15897 | 0.44787 | 0.26247 |
| C | 1.79364 | -1.48464 | -0.15123 |
| C | 4.87177 | -0.32899 | 1.18084 |
| C | 4.81424 | 0.88071 | -0.89692 |
| C | 6.20426 | -0.66887 | 0.94903 |
| C | 6.14386 | 0.54193 | -1.13262 |
| C | 6.84357 | -0.23551 | -0.20988 |
| C | 1.34522 | -3.22381 | 1.39656 |
| H | -4.32017 | -0.79255 | 1.0814 |
| H | -2.81596 | -1.68842 | 1.19512 |
| H | -2.08418 | -0.78704 | -1.00699 |
| H | -3.60686 | 0.08468 | -1.17546 |
| H | -5.02763 | -1.5068 | -1.96995 |
| H | -5.92684 | -2.95434 | -1.51979 |
| H | -6.12043 | -1.47004 | -0.57986 |
| H | -5.33868 | -4.29972 | 0.50797 |
| H | -5.57566 | -2.86917 | 1.52015 |
| H | -4.05735 | -3.77266 | 1.60748 |
| H | -2.81939 | -2.80803 | -1.83794 |
| H | -2.39374 | -3.68404 | -0.36109 |
| H | -3.70033 | -4.26894 | -1.39678 |
| H | -2.22322 | 2.00471 | -1.40609 |
| H | -3.04538 | 1.21684 | 0.88736 |
| H | -0.93791 | 3.85094 | -0.28694 |
| H | -1.05786 | 3.00688 | 1.24689 |
| H | 1.93634 | 0.19433 | -1.42848 |
| H | -0.21049 | 0.10868 | 0.61794 |
| H | 2.45097 | 0.64804 | 1.55485 |
| H | 2.54568 | 1.86611 | 0.28329 |
| H | 4.38204 | -0.66466 | 2.08969 |
| H | 4.27954 | 1.49112 | -1.61798 |
| H | 6.74184 | -1.26925 | 1.6747 |
| H | 6.63532 | 0.88695 | -2.03551 |
| H | -3.59265 | 5.47919 | 1.13126 |
| H | 7.87945 | -0.49811 | -0.3925 |
| H | 0.92099 | -3.29944 | 2.39522 |
| H | 2.37268 | -3.58894 | 1.38462 |
| H | 0.75287 | -3.79567 | 0.68128 |
| Zn | 3.47773 | -3.29326 | 0.59742 |

**Cartesian coordinates of Neotame-Zn^2+^ complex optimized using 6-311G (d, p) basis set in gas phase for position 3**

| O | 0.3043 | 2.00249 | -1.84652 |
| --- | --- | --- | --- |
| O | 1.31035 | -1.82051 | 1.06726 |
| O | -2.71516 | 5.11528 | 0.93723 |
| O | -3.97475 | 3.30867 | 0.50667 |
| O | 2.23854 | -2.28066 | -0.93564 |
| N | -2.29949 | 0.78834 | 0.34856 |
| N | 0.33722 | 0.47166 | -0.1545 |
| C | -4.25005 | -2.53774 | -0.18996 |
| C | -3.56517 | -1.32095 | 0.48411 |
| C | -2.88808 | -0.30808 | -0.44099 |
| C | -5.39403 | -2.08657 | -1.11889 |
| C | -4.83939 | -3.42081 | 0.92712 |
| C | -3.22983 | -3.36726 | -0.99308 |
| C | -1.67143 | 1.82395 | -0.47187 |
| C | -1.55939 | 3.15757 | 0.28304 |
| C | -0.2435 | 1.43568 | -0.91132 |
| C | 1.70363 | 0.02131 | -0.37849 |
| C | 2.71067 | 0.80812 | 0.50528 |
| C | -2.88451 | 3.82562 | 0.56777 |
| C | 4.15897 | 0.44787 | 0.26247 |
| C | 1.79364 | -1.48464 | -0.15123 |
| C | 4.87177 | -0.32899 | 1.18084 |
| C | 4.81424 | 0.88071 | -0.89692 |
| C | 6.20426 | -0.66887 | 0.94903 |
| C | 6.14386 | 0.54193 | -1.13262 |
| C | 6.84357 | -0.23551 | -0.20988 |
| C | 1.34522 | -3.22381 | 1.39656 |
| H | -4.32017 | -0.79255 | 1.0814 |
| H | -2.81596 | -1.68842 | 1.19512 |
| H | -2.08418 | -0.78704 | -1.00699 |
| H | -3.60686 | 0.08468 | -1.17546 |
| H | -5.02763 | -1.5068 | -1.96995 |
| H | -5.92684 | -2.95434 | -1.51979 |
| H | -6.12043 | -1.47004 | -0.57986 |
| H | -5.33868 | -4.29972 | 0.50797 |
| H | -5.57566 | -2.86917 | 1.52015 |
| H | -4.05735 | -3.77266 | 1.60748 |
| H | -2.81939 | -2.80803 | -1.83794 |
| H | -2.39374 | -3.68404 | -0.36109 |
| H | -3.70033 | -4.26894 | -1.39678 |
| H | -2.22322 | 2.00471 | -1.40609 |
| H | -3.04538 | 1.21684 | 0.88736 |
| H | -0.93791 | 3.85094 | -0.28694 |
| H | -1.05786 | 3.00688 | 1.24689 |
| H | 1.93634 | 0.19433 | -1.42848 |
| H | -0.21049 | 0.10868 | 0.61794 |
| H | 2.45097 | 0.64804 | 1.55485 |
| H | 2.54568 | 1.86611 | 0.28329 |
| H | 4.38204 | -0.66466 | 2.08969 |
| H | 4.27954 | 1.49112 | -1.61798 |
| H | 6.74184 | -1.26925 | 1.6747 |
| H | 6.63532 | 0.88695 | -2.03551 |
| H | -3.59265 | 5.47919 | 1.13126 |
| H | 7.87945 | -0.49811 | -0.3925 |
| H | 0.92099 | -3.29944 | 2.39522 |
| H | 2.37268 | -3.58894 | 1.38462 |
| H | 0.75287 | -3.79567 | 0.68128 |
| Zn | 1.63835 | 3.82181 | 0.05218 |

**Cartesian coordinates of Neotame-Zn^2+^ complex optimized using 6-311G (d, p) basis set in water for position 1**

| O | 0.3043 | 2.00249 | -1.84652 |
| --- | --- | --- | --- |
| O | 1.31035 | -1.82051 | 1.06726 |
| O | -2.71516 | 5.11528 | 0.93723 |
| O | -3.97475 | 3.30867 | 0.50667 |
| O | 2.23854 | -2.28066 | -0.93564 |
| N | -2.29949 | 0.78834 | 0.34856 |
| N | 0.33722 | 0.47166 | -0.1545 |
| C | -4.25005 | -2.53774 | -0.18996 |
| C | -3.56517 | -1.32095 | 0.48411 |
| C | -2.88808 | -0.30808 | -0.44099 |
| C | -5.39403 | -2.08657 | -1.11889 |
| C | -4.83939 | -3.42081 | 0.92712 |
| C | -3.22983 | -3.36726 | -0.99308 |
| C | -1.67143 | 1.82395 | -0.47187 |
| C | -1.55939 | 3.15757 | 0.28304 |
| C | -0.2435 | 1.43568 | -0.91132 |
| C | 1.70363 | 0.02131 | -0.37849 |
| C | 2.71067 | 0.80812 | 0.50528 |
| C | -2.88451 | 3.82562 | 0.56777 |
| C | 4.15897 | 0.44787 | 0.26247 |
| C | 1.79364 | -1.48464 | -0.15123 |
| C | 4.87177 | -0.32899 | 1.18084 |
| C | 4.81424 | 0.88071 | -0.89692 |
| C | 6.20426 | -0.66887 | 0.94903 |
| C | 6.14386 | 0.54193 | -1.13262 |
| C | 6.84357 | -0.23551 | -0.20988 |
| C | 1.34522 | -3.22381 | 1.39656 |
| H | -4.32017 | -0.79255 | 1.0814 |
| H | -2.81596 | -1.68842 | 1.19512 |
| H | -2.08418 | -0.78704 | -1.00699 |
| H | -3.60686 | 0.08468 | -1.17546 |
| H | -5.02763 | -1.5068 | -1.96995 |
| H | -5.92684 | -2.95434 | -1.51979 |
| H | -6.12043 | -1.47004 | -0.57986 |
| H | -5.33868 | -4.29972 | 0.50797 |
| H | -5.57566 | -2.86917 | 1.52015 |
| H | -4.05735 | -3.77266 | 1.60748 |
| H | -2.81939 | -2.80803 | -1.83794 |
| H | -2.39374 | -3.68404 | -0.36109 |
| H | -3.70033 | -4.26894 | -1.39678 |
| H | -2.22322 | 2.00471 | -1.40609 |
| H | -3.04538 | 1.21684 | 0.88736 |
| H | -0.93791 | 3.85094 | -0.28694 |
| H | -1.05786 | 3.00688 | 1.24689 |
| H | 1.93634 | 0.19433 | -1.42848 |
| H | -0.21049 | 0.10868 | 0.61794 |
| H | 2.45097 | 0.64804 | 1.55485 |
| H | 2.54568 | 1.86611 | 0.28329 |
| H | 4.38204 | -0.66466 | 2.08969 |
| H | 4.27954 | 1.49112 | -1.61798 |
| H | 6.74184 | -1.26925 | 1.6747 |
| H | 6.63532 | 0.88695 | -2.03551 |
| H | -3.59265 | 5.47919 | 1.13126 |
| H | 7.87945 | -0.49811 | -0.3925 |
| H | 0.92099 | -3.29944 | 2.39522 |
| H | 2.37268 | -3.58894 | 1.38462 |
| H | 0.75287 | -3.79567 | 0.68128 |
| Zn | -5.67578 | 4.36842 | 1.13654 |

**Cartesian coordinates of Neotame-Zn^2+^ complex optimized using 6-311G (d, p) basis set in water for position 2**

| O | 0.3043 | 2.00249 | -1.84652 |
| --- | --- | --- | --- |
| O | 1.31035 | -1.82051 | 1.06726 |
| O | -2.71516 | 5.11528 | 0.93723 |
| O | -3.97475 | 3.30867 | 0.50667 |
| O | 2.23854 | -2.28066 | -0.93564 |
| N | -2.29949 | 0.78834 | 0.34856 |
| N | 0.33722 | 0.47166 | -0.1545 |
| C | -4.25005 | -2.53774 | -0.18996 |
| C | -3.56517 | -1.32095 | 0.48411 |
| C | -2.88808 | -0.30808 | -0.44099 |
| C | -5.39403 | -2.08657 | -1.11889 |
| C | -4.83939 | -3.42081 | 0.92712 |
| C | -3.22983 | -3.36726 | -0.99308 |
| C | -1.67143 | 1.82395 | -0.47187 |
| C | -1.55939 | 3.15757 | 0.28304 |
| C | -0.2435 | 1.43568 | -0.91132 |
| C | 1.70363 | 0.02131 | -0.37849 |
| C | 2.71067 | 0.80812 | 0.50528 |
| C | -2.88451 | 3.82562 | 0.56777 |
| C | 4.15897 | 0.44787 | 0.26247 |
| C | 1.79364 | -1.48464 | -0.15123 |
| C | 4.87177 | -0.32899 | 1.18084 |
| C | 4.81424 | 0.88071 | -0.89692 |
| C | 6.20426 | -0.66887 | 0.94903 |
| C | 6.14386 | 0.54193 | -1.13262 |
| C | 6.84357 | -0.23551 | -0.20988 |
| C | 1.34522 | -3.22381 | 1.39656 |
| H | -4.32017 | -0.79255 | 1.0814 |
| H | -2.81596 | -1.68842 | 1.19512 |
| H | -2.08418 | -0.78704 | -1.00699 |
| H | -3.60686 | 0.08468 | -1.17546 |
| H | -5.02763 | -1.5068 | -1.96995 |
| H | -5.92684 | -2.95434 | -1.51979 |
| H | -6.12043 | -1.47004 | -0.57986 |
| H | -5.33868 | -4.29972 | 0.50797 |
| H | -5.57566 | -2.86917 | 1.52015 |
| H | -4.05735 | -3.77266 | 1.60748 |
| H | -2.81939 | -2.80803 | -1.83794 |
| H | -2.39374 | -3.68404 | -0.36109 |
| H | -3.70033 | -4.26894 | -1.39678 |
| H | -2.22322 | 2.00471 | -1.40609 |
| H | -3.04538 | 1.21684 | 0.88736 |
| H | -0.93791 | 3.85094 | -0.28694 |
| H | -1.05786 | 3.00688 | 1.24689 |
| H | 1.93634 | 0.19433 | -1.42848 |
| H | -0.21049 | 0.10868 | 0.61794 |
| H | 2.45097 | 0.64804 | 1.55485 |
| H | 2.54568 | 1.86611 | 0.28329 |
| H | 4.38204 | -0.66466 | 2.08969 |
| H | 4.27954 | 1.49112 | -1.61798 |
| H | 6.74184 | -1.26925 | 1.6747 |
| H | 6.63532 | 0.88695 | -2.03551 |
| H | -3.59265 | 5.47919 | 1.13126 |
| H | 7.87945 | -0.49811 | -0.3925 |
| H | 0.92099 | -3.29944 | 2.39522 |
| H | 2.37268 | -3.58894 | 1.38462 |
| H | 0.75287 | -3.79567 | 0.68128 |
| Zn | 3.47773 | -3.29326 | 0.59742 |

**Cartesian coordinates of Neotame-Zn^2+^ complex optimized using 6-311G (d, p) basis set in water for position 3**

| O | 0.3043 | 2.00249 | -1.84652 |
| --- | --- | --- | --- |
| O | 1.31035 | -1.82051 | 1.06726 |
| O | -2.71516 | 5.11528 | 0.93723 |
| O | -3.97475 | 3.30867 | 0.50667 |
| O | 2.23854 | -2.28066 | -0.93564 |
| N | -2.29949 | 0.78834 | 0.34856 |
| N | 0.33722 | 0.47166 | -0.1545 |
| C | -4.25005 | -2.53774 | -0.18996 |
| C | -3.56517 | -1.32095 | 0.48411 |
| C | -2.88808 | -0.30808 | -0.44099 |
| C | -5.39403 | -2.08657 | -1.11889 |
| C | -4.83939 | -3.42081 | 0.92712 |
| C | -3.22983 | -3.36726 | -0.99308 |
| C | -1.67143 | 1.82395 | -0.47187 |
| C | -1.55939 | 3.15757 | 0.28304 |
| C | -0.2435 | 1.43568 | -0.91132 |
| C | 1.70363 | 0.02131 | -0.37849 |
| C | 2.71067 | 0.80812 | 0.50528 |
| C | -2.88451 | 3.82562 | 0.56777 |
| C | 4.15897 | 0.44787 | 0.26247 |
| C | 1.79364 | -1.48464 | -0.15123 |
| C | 4.87177 | -0.32899 | 1.18084 |
| C | 4.81424 | 0.88071 | -0.89692 |
| C | 6.20426 | -0.66887 | 0.94903 |
| C | 6.14386 | 0.54193 | -1.13262 |
| C | 6.84357 | -0.23551 | -0.20988 |
| C | 1.34522 | -3.22381 | 1.39656 |
| H | -4.32017 | -0.79255 | 1.0814 |
| H | -2.81596 | -1.68842 | 1.19512 |
| H | -2.08418 | -0.78704 | -1.00699 |
| H | -3.60686 | 0.08468 | -1.17546 |
| H | -5.02763 | -1.5068 | -1.96995 |
| H | -5.92684 | -2.95434 | -1.51979 |
| H | -6.12043 | -1.47004 | -0.57986 |
| H | -5.33868 | -4.29972 | 0.50797 |
| H | -5.57566 | -2.86917 | 1.52015 |
| H | -4.05735 | -3.77266 | 1.60748 |
| H | -2.81939 | -2.80803 | -1.83794 |
| H | -2.39374 | -3.68404 | -0.36109 |
| H | -3.70033 | -4.26894 | -1.39678 |
| H | -2.22322 | 2.00471 | -1.40609 |
| H | -3.04538 | 1.21684 | 0.88736 |
| H | -0.93791 | 3.85094 | -0.28694 |
| H | -1.05786 | 3.00688 | 1.24689 |
| H | 1.93634 | 0.19433 | -1.42848 |
| H | -0.21049 | 0.10868 | 0.61794 |
| H | 2.45097 | 0.64804 | 1.55485 |
| H | 2.54568 | 1.86611 | 0.28329 |
| H | 4.38204 | -0.66466 | 2.08969 |
| H | 4.27954 | 1.49112 | -1.61798 |
| H | 6.74184 | -1.26925 | 1.6747 |
| H | 6.63532 | 0.88695 | -2.03551 |
| H | -3.59265 | 5.47919 | 1.13126 |
| H | 7.87945 | -0.49811 | -0.3925 |
| H | 0.92099 | -3.29944 | 2.39522 |
| H | 2.37268 | -3.58894 | 1.38462 |
| H | 0.75287 | -3.79567 | 0.68128 |
| Zn | 1.63835 | 3.82181 | 0.05218 |


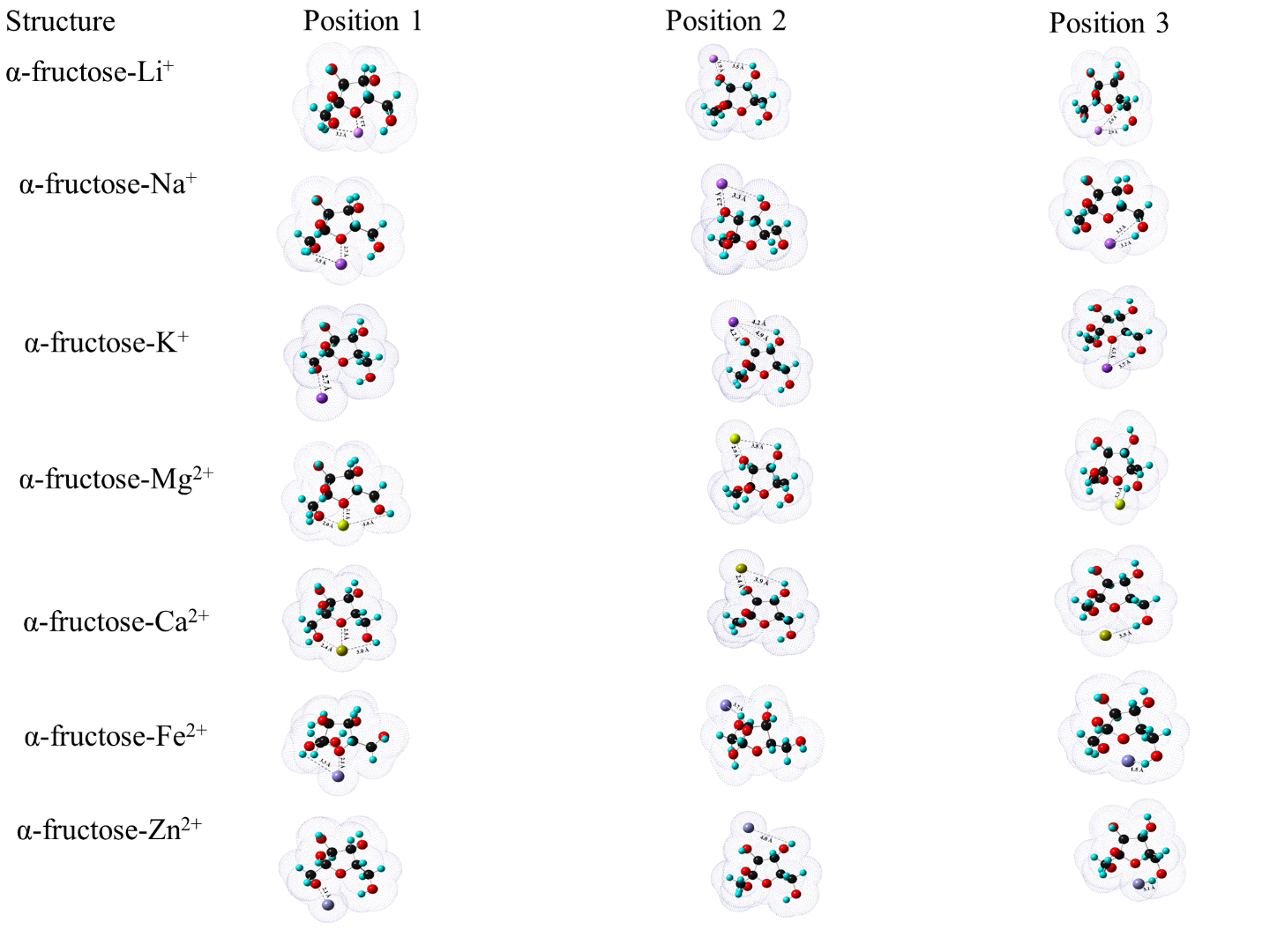


Fig. S1.


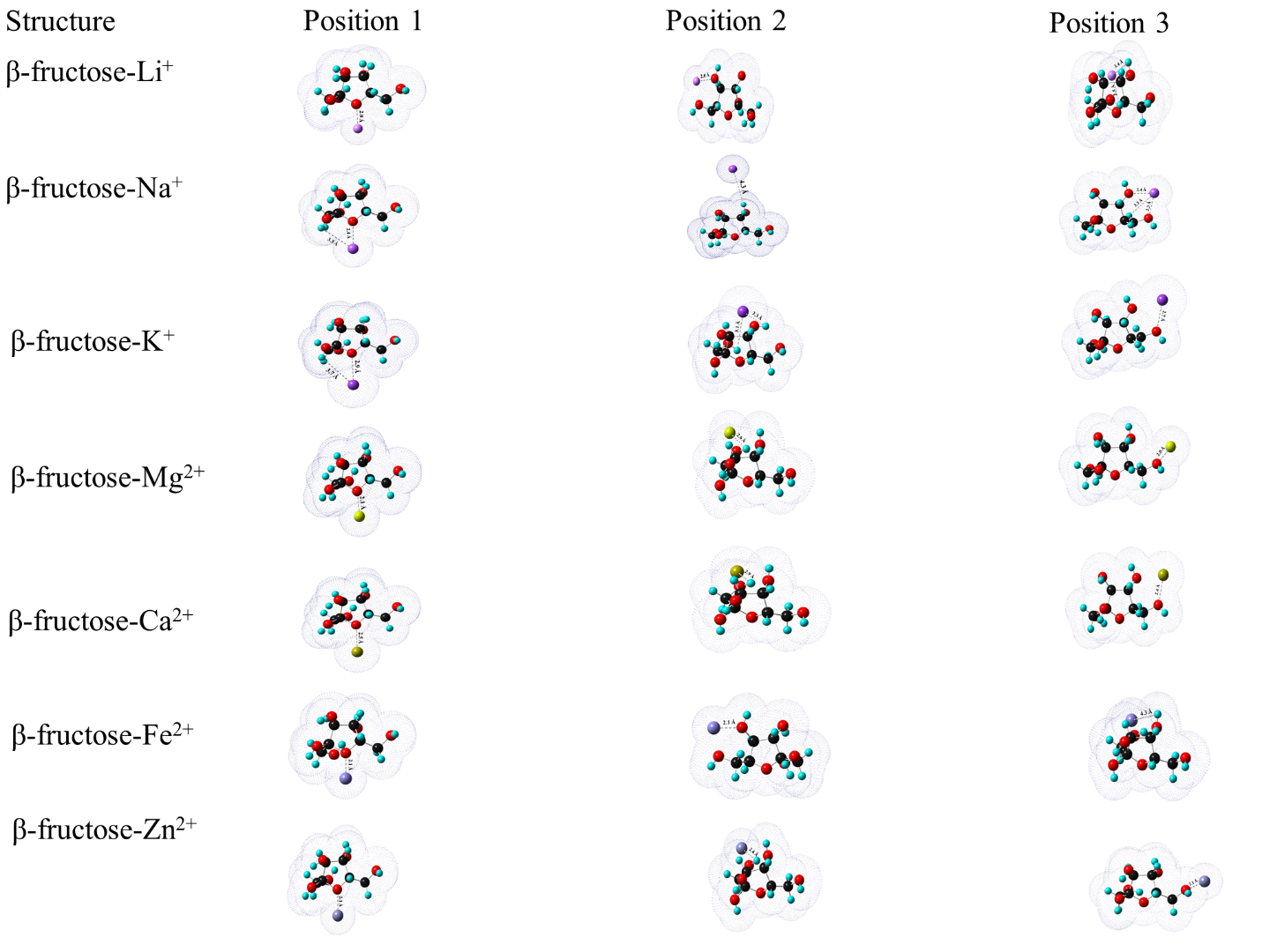


Fig. S2.


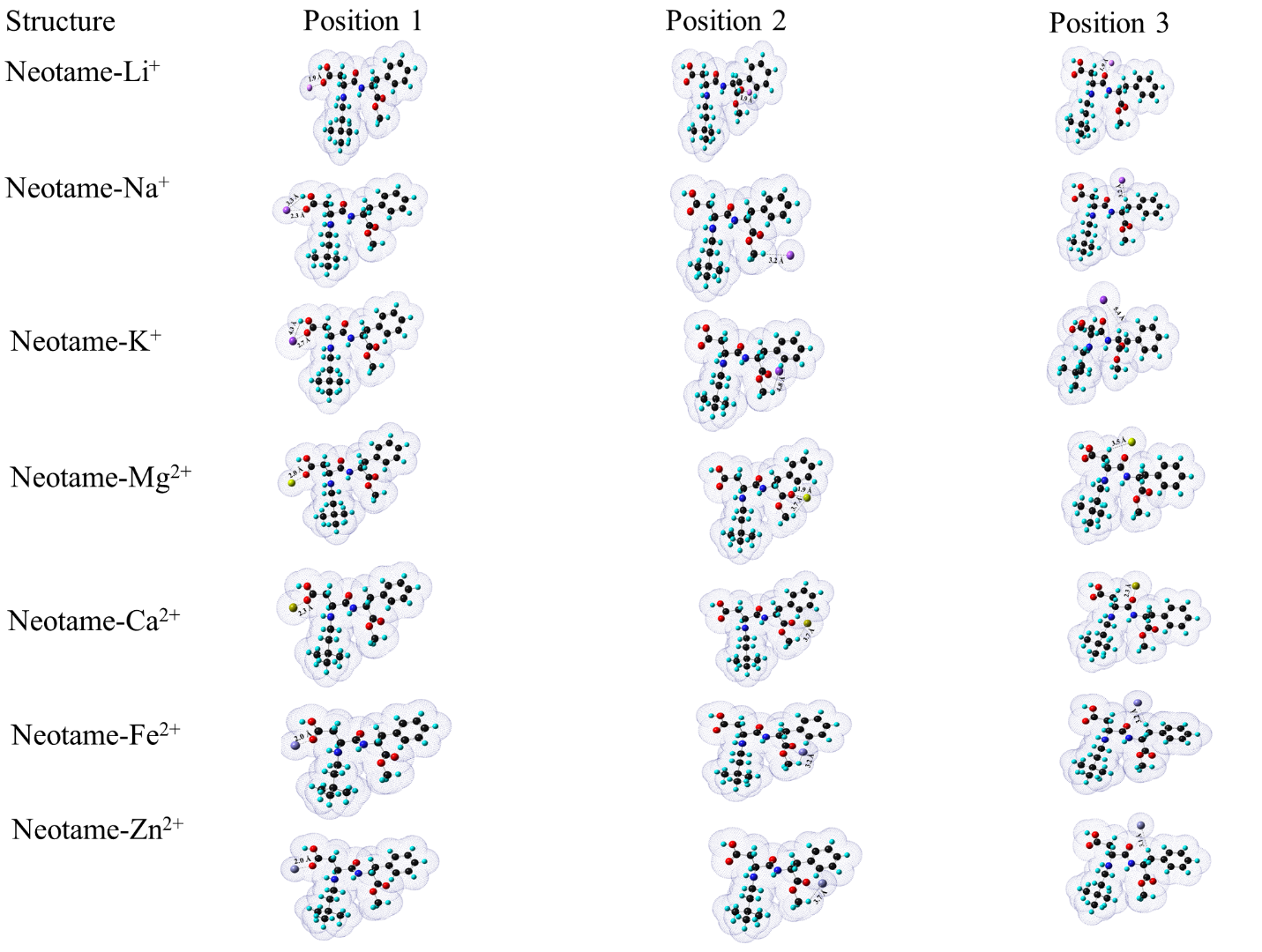


Fig. S3.


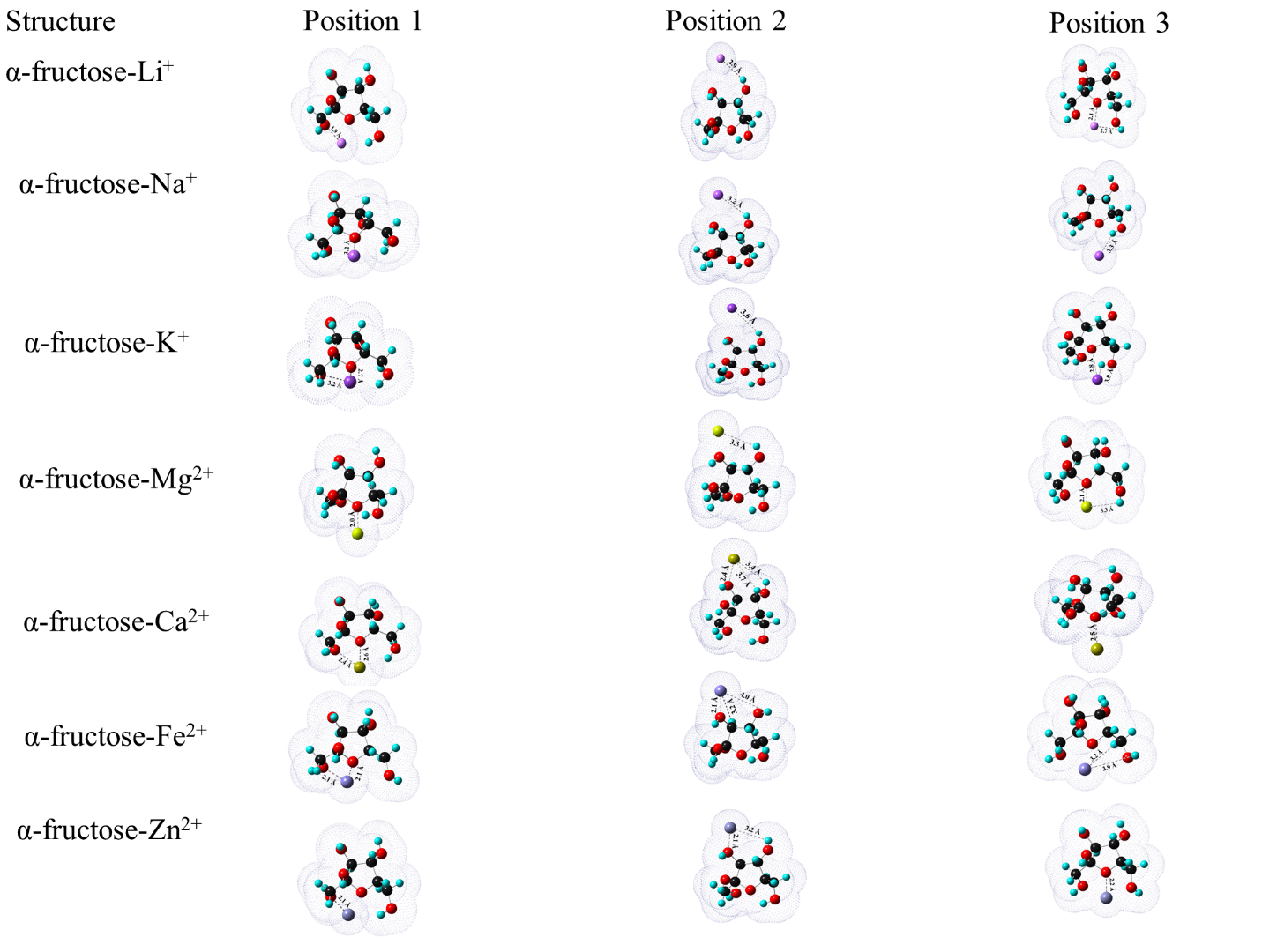


Fig. S4.


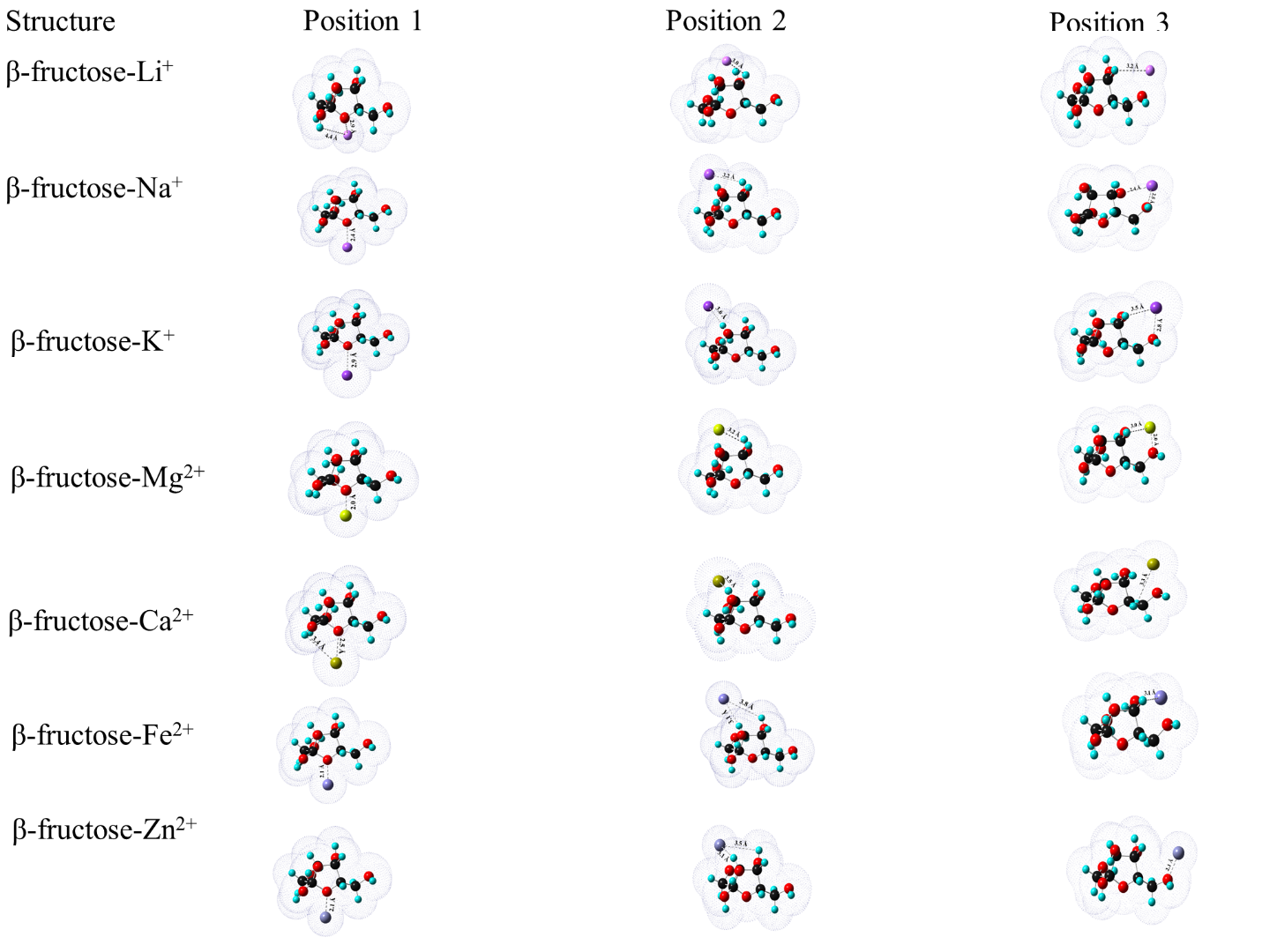


Fig. S5.


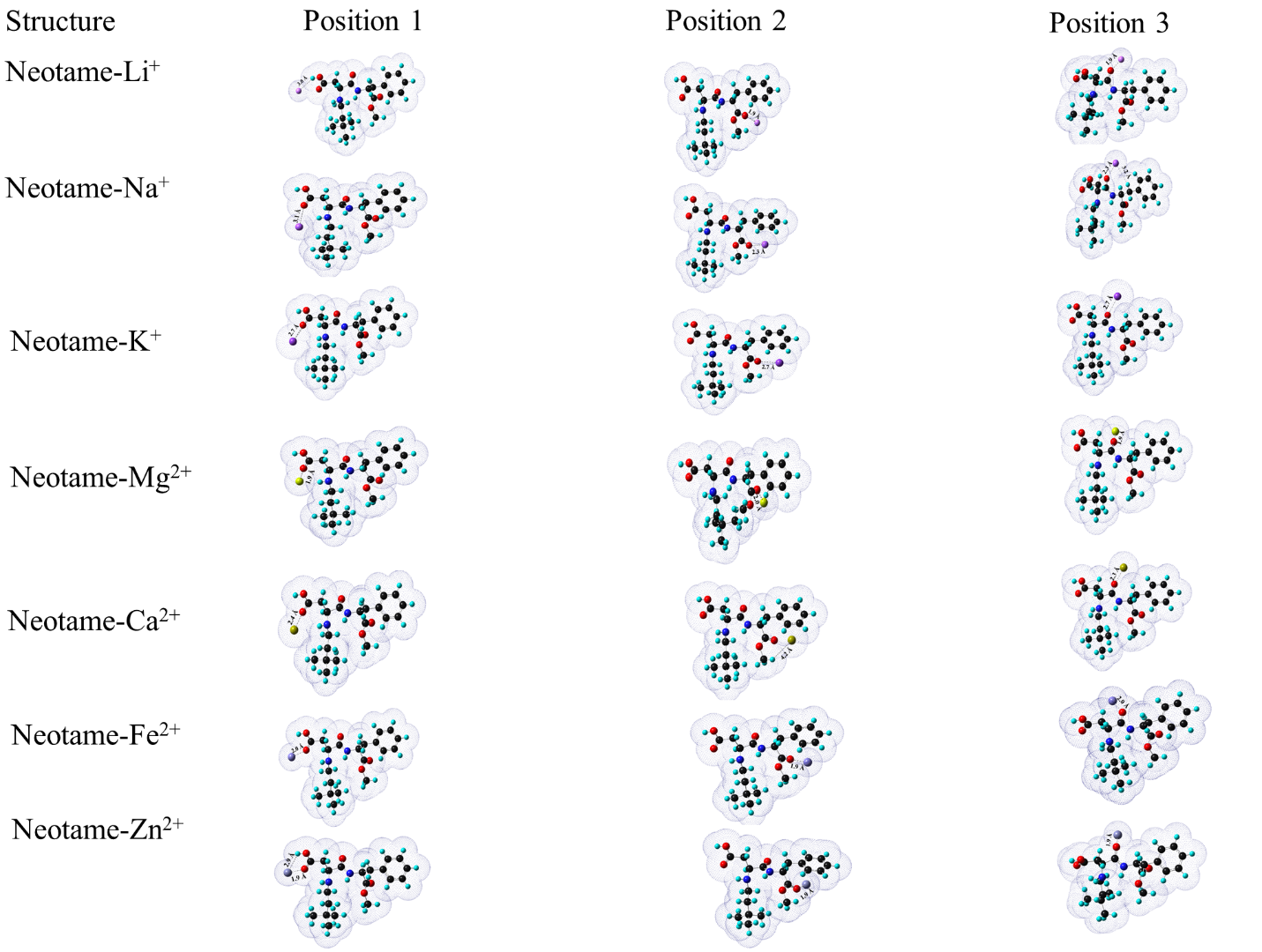


Fig. S6.


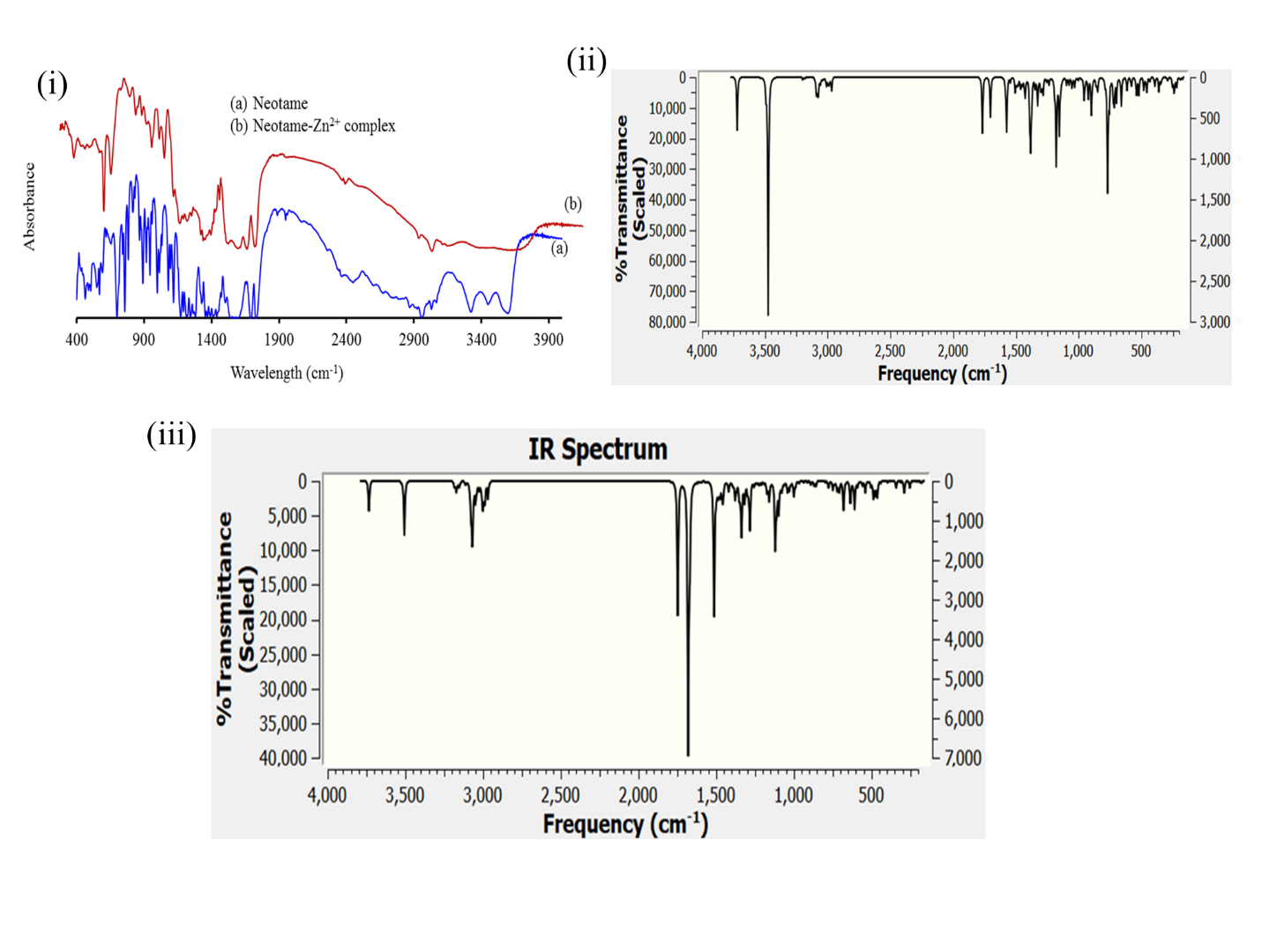


Fig. S7.


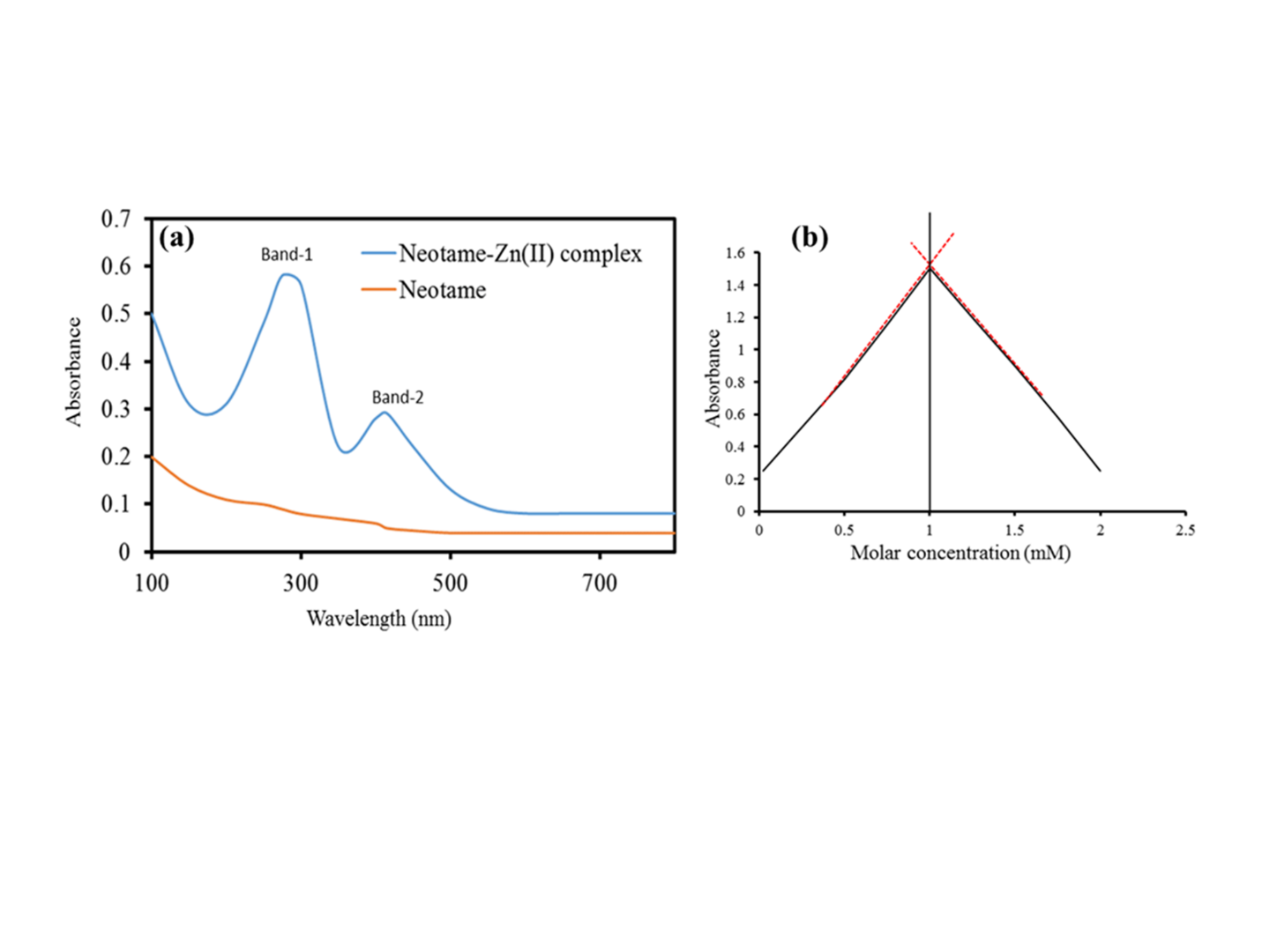
Fig. S8.
